# Inefficient quality control of ribosome stalling during APP synthesis generates CAT-tailed species that precipitate hallmarks of Alzheimer’s disease

**DOI:** 10.1186/s40478-021-01268-6

**Published:** 2021-10-18

**Authors:** Suman Rimal, Yu Li, Rasika Vartak, Ji Geng, Ishaq Tantray, Shuangxi Li, Sungun Huh, Hannes Vogel, Charles Glabe, Lea T. Grinberg, Salvatore Spina, William W. Seeley, Su Guo, Bingwei Lu

**Affiliations:** 1 Department of Pathology, Stanford University School of Medicine, Stanford, CA 94305 USA; 2 Department of Molecular Biology and Biochemistry, University of California, Irvine, CA USA; 3 Memory and Aging Center, Department of Neurology and Department of Pathology, University of California, San Francisco, CA 94158 USA; 4 Department of Bioengineering and Therapeutic Sciences, Programs in Human Genetics and Biological Sciences, Eli and Edythe Broad Center of Regeneration Medicine and Stem Cell Research, University of California, San Francisco, CA USA

**Keywords:** Alzheimer’s disease, Amyloid precursor protein C-terminal fragment (APP.C99), Ribosome stalling, Ribosome-associated quality control, CAT-tailing, Proteostasis

## Abstract

Amyloid precursor protein (APP) metabolism is central to Alzheimer’s disease (AD) pathogenesis, but the key etiological driver remains elusive. Recent failures of clinical trials targeting amyloid-β (Aβ) peptides, the proteolytic fragments of amyloid precursor protein (APP) that are the main component of amyloid plaques, suggest that the proteostasis-disrupting, key pathogenic species remain to be identified. Previous studies suggest that APP C-terminal fragment (APP.C99) can cause disease in an Aβ-independent manner. The mechanism of APP.C99 pathogenesis is incompletely understood. We used *Drosophila* models expressing APP.C99 with the native ER-targeting signal of human APP, expressing full-length human APP only, or co-expressing full-length human APP and β-secretase (BACE), to investigate mechanisms of APP.C99 pathogenesis. Key findings are validated in mammalian cell culture models, mouse 5xFAD model, and postmortem AD patient brain materials. We find that ribosomes stall at the ER membrane during co-translational translocation of APP.C99, activating ribosome-associated quality control (RQC) to resolve ribosome collision and stalled translation. Stalled APP.C99 species with C-terminal extensions (CAT-tails) resulting from inadequate RQC are prone to aggregation, causing endolysosomal and autophagy defects and seeding the aggregation of amyloid β peptides, the main component of amyloid plaques. Genetically removing stalled and CAT-tailed APP.C99 rescued proteostasis failure, endolysosomal/autophagy dysfunction, neuromuscular degeneration, and cognitive deficits in AD models. Our finding of RQC factor deposition at the core of amyloid plaques from AD brains further supports the central role of defective RQC of ribosome collision and stalled translation in AD pathogenesis. These findings demonstrate that amyloid plaque formation is the consequence and manifestation of a deeper level proteostasis failure caused by inadequate RQC of translational stalling and the resultant aberrantly modified APP.C99 species, previously unrecognized etiological drivers of AD and newly discovered therapeutic targets.

**Supplementary Information:**

The online version contains supplementary material available at 10.1186/s40478-021-01268-6.

## Introduction

Proteostasis denotes a cellular state in which protein synthesis, folding, and degradation are maintained at a homeostatic state such that an intact yet dynamic proteome is preserved [1, 2]. It is generally believed that the cellular capacity to preserve proteostasis declines with age, contributing to the pathogenesis of age-related diseases [3]. Proteostasis failure manifested as formation of aberrant protein aggregates, including the amyloid plaques composed of the β-amyloid (Aβ) peptide in AD [4, 5], is a defining feature of age-related neurodegenerative diseases [6–8]. Aβ is one of the metabolites of amyloid precursor protein (APP). Although the physiological function of APP remains incompletely understood [9], Aβ is known to be derived from the trafficking and processing of APP through the amyloidogenic pathway [10–12], whereby APP is cleaved by β-secretase (BACE) to generate soluble APP beta protein (sAPPβ) and APP.C99, and APP.C99 cleaved by γ-secretase to release Aβ and APP intracellular domain (AICD). The facts that heterozygous mutations in the APP and the presenilin (PSEN1 and PSEN2) genes are sufficient to cause rare, early onset forms of AD [13], and that PSEN1/PSEN2 are components of the γ-secretase complex [14] provide compelling evidence that APP abnormality is central to AD pathogenesis [15], at least in the rare familial cases. The origin of proteostasis failure and protein aggregate formation in the more common sporadic cases, however, is still enigmatic. Studies in various experimental systems support the idea that cellular co-factors play important roles in “seeding” protein aggregate formation [16–18]. But the molecular nature of the initial seeding-activities and cellular mechanisms governing their formation remain largely undefined.

Although previous studies of proteostasis failure in neurodegenerative disease focused heavily on post-synthesis, mature proteins, recent studies provide compelling evidence that problems of proteostasis can begin with nascent peptide chains (NPCs) still associated with translating ribosomes, necessitating the deployment of ribosome-associated quality control (RQC) pathway to handle faculty translation products [19–21]. During translation, ribosome slowdown and stalling can occur for a number of reasons. Some are functional and serve to facilitate cellular dynamics, such as co-translational protein folding, subcellular protein targeting, and mRNA localization. Others are detrimental and can be triggered by damaged mRNAs, mRNA secondary structures, insufficient supply of aminoacyl-tRNAs, inefficient ribosome termination/recycling, or environmental stress [19–21]. Unmitigated ribosome stalling can result in ribosome collision, which is sensed by the cell as a proxy for aberrant translation and triggers ribosome-mediated quality control [22]. A key factor involved is the ubiquitin ligase ZNF598, which recognizes the distinct 40S-40S interface characterizing collided ribosomes and ubiquitinates specific 40S subunit proteins [23, 24]. This triggers a series of downstream quality control events, ribosome subunit separation and recycling by ABCE1 [25], degradation by the Ltn1/VCP/NEMF complex of the NPCs still attached to the 60S subunit [26, 27], and mRNA degradation. As the QC machinery is typically sub-stoichiometric to ribosomes [28], inefficient QC of the NPC-60S complex can happen and may lead to the formation of aberrant NPCs containing C-terminal Ala and Thr additions (CAT-tails) [29]. Although CAT-tails are hypothesized to function as degrons to promote the degradation of stalled NPCs [30], their accumulation can be detrimental to proteostasis [31–33]. So far studies of ribosome stalling, RQC, and CAT-tailing have been largely limited to single cell organisms and using artificial substrates. Whether they contribute to proteostasis failure in major diseases like AD is not known.

Here we report a previously unrecognized role of stalled translation of APP at the ER translocon, arguably the earliest stage in APP’s lifecycle, in generating cytotoxic species of APP.C99. Our studies in mammalian cell culture, *Drosophila* and mouse AD models, and post-mortem AD patient brain samples support the notion that the proper translation and biogenesis of APP.C99 depend on RQC activity, the inadequacy of which can result in ribosome collision  and accumulation of translationally stalled and CAT-tailed APP.C99 species that impair the endolysosomal and autophagy systems and seed the formation of amyloid plaques, causing proteostasis failure and AD pathogenesis. Our results establish defective RQC of stalled APP.C99 translation and generation of aberrantly modified APP.C99 as potentially one of the earliest pathogenic events of AD.

## Materials and methods

### Chemical sources

Sucrose (#S0389), Chloroquine (#C6628), Quinine (#6119–47-7), Tris base (#11,814,273,001), Glycine (#G8898), SDS (#L3771), Triton™ X-100 (#T9284), EDTA (#E9884), EGTA (#3889), Dimethyl sulfoxide (#D8418), Thio-T (#T3516), HHT (#SML1091), Emitine (#E2375) were purchased from Sigma-Aldrich. Anisomycin (#S7409) was purchased from SelleckChem. DMEM, high glucose, GlutaMAX Supplement (#10,566,016) was purchased from GIBCO. Complete™, Mini, EDTA-free Protease Inhibitor Cocktail (#11,836,170,001) was purchased form Roche. Lipofectamine 3000 (#L3000015), Lipofectamine RNAi-MAC (#13,778,150), Puromycin (ant-pr-1) were purchased from Invitrogen. Pierce 16% Formaldehyde (w/v), Methanol-free (#28,908), RPMI-1640 (#11,845,085), RNAse A (#12,091,021) were purchased from ThermoFisher.

### *Drosophila* genetics and husbandry

We used wild type *w*^*1118*^ as control. We obtained the following lines from the Bloomington *Drosophila* Stock Center: *elav-GAL4* (B8765), *UAS-APP.C99* (B33783), *UAS-APP* (B6700), *UAS-APP;UAS-BACE* (B33798), *UAS-Rab5-GFP* (B43336), *UAS-KDEL-GFP* (B9898), *UAS-LAMP1-GFP* (B42714), *UAS-Rab5*-WT (B43336), *UAS-Rab5*-CA (B43335), *UAS-Rab5*-DN (B9772), *UAS-Fip200* (B91226), *UAS-ABCE1-RNAi* (B31601), *UAS-Lcp2-C99* (B33785), *UAS-ZNF598-RNAi* (B61288), *UAS-Ltn1* (B30116). We obtained *UAS-Clbn-RNAi* (v103351) from Vienna Drosophila Stock Center. We obtained *UAS-ABCE1* (F001097), *UAS-ZNF598* (F001909), *UAS-Pelo* (F003036), *UAS-Vms1* (F002500) from FLYORF. T. Littleton provided *MHC-GAL4*, S. Birman provided *TH-GAL4*, F. Kawasaki provided *UAS-mito-GCaMP*, N. Bonini provided *UAS-HspA1L*, Eric Baehrecke provided *UAS-ATG1*, Paul Taylor provided *UAS-VCP*. The indicated *UAS* RNAi and OE fly lines were crossed to *Mhc-Gal4* or *elav-Gal4* driver lines for muscle or pan-neuronal expression, respectively. Fly culture and crosses were performed according to standard procedures. Adult flies were generally raised at 25 °C and with 12/12 h dark/light cycles. Fly food was prepared with a standard receipt (Water, 17 L; Agar, 93 g; Cornmeal, 1,716 g; Brewer’s yeast extract, 310 g; Sucrose, 517 g; Dextrose, 1033 g).

### Climbing assay

10–20 male flies were transferred to a clean empty food vial. The flies were then allowed to get accustomed to the new environment for 3–4 min and subsequently measured for bang-induced vertical climbing distance. The performed was scored as percentage of flies crossing the 8 cm mark within 12 s. Each experiment was carried out ≥ 4 times.

### Lifespan analysis

Flies were reared in vials containing cornmeal medium. Flies were anesthetized using CO_2_ and collected at a density of 20 male flies/vials. All flies were kept at humidified, 12 h on/off light cycle at 25 °C. Flies were flipped into fresh vial every 3 days and scored for death. Each set of experiment was carried out ≥ 4 times.

### Extraction of fly proteins for western blot analysis

Around 5 fly thoraces were homogenized in 80 µl of either regular lysis buffer (50 mM Tris–HCl, 150 mM NaCl, 1% Triton X100, protease inhibitors) or Urea buffer (6 M Urea, 50 mM Tris–HCl, 150 mM NaCl, 0.1% Triton X100, protease inhibitors) on ice. Each sample was homogenized by a mechanical homogenizer for 30 secs. The homogenized samples were incubated on ice for 30 min before centrifuging at 15,000 rpm for 20 min at 4 °C. 30 µl of supernatant was mixed with 10 µl of 4 × Lammaelli buffer (BioRad #161–0747) and boiled for 5 min at 100 °C. The protein lysate was cooled, centrifuged and loaded onto 4–12% Bis–Tris gel (Invitrogen #NP0321) or 16% Tricine gel (Invitrogen #EC66955) with 1 × MES (Invitrogen #NP0002) as running buffer.

### Aversive taste memory

Taste memory assay was performed as described previously [34], with slight modifications. Briefly, one-week old flies were starved for 12–18 h in an empty vial on wet Kimwipe paper. Flies were later anesthetized using ice and fixed on a glass slides by applying nail polish to their wings. 10–15 flies were used for each set of experiment. The flies were then incubated in a humid chamber for 2 h to allow them to recover from the procedure. The experiment was divided into three phases. The first phase is the pretest, when the flies were presented with 500 mM sucrose stimuli (attractive tastant) to their legs using a Kimwipe wick. Flies that showed positive proboscis extension to the stimulus were used for the next phases. The second phase was the training phase, when the flies were presented with 500 mM sucrose stimuli at their legs while simultaneously being punished by applying 10 mM quinine (aversive tastant) on their extended proboscis. Training was repeated 15 times for each fly. The last phase is the test phase when the flies were given 500 mM sucrose to their legs at different time intervals (0, 5, 15, 30, 45, and 60 min), and the proboscis extension response was recorded. Each experiment was carried out ≥ 4 times.

### Immunohistochemistry

We performed immunostaining analysis of adult fly muscle as previously described [35]. Briefly, fly thoraxes were dissected and fixed with 4% paraformaldehyde (Electron Microscopy Sciences, cat. no. 15710) in phosphate buffered saline and 0.3% Triton X-100 (PBS-T). The tissues were then washed three times with PBS-T. The samples were incubated for 30 min at room temperature in blocking buffer: 0.5% goat serum in PBS-T. The indicated primary antibodies (anti-Ubiquitin, Abcam ab140601, 1:1000; anti-Rab5, Abcam ab31261, 1:1000; anti-P62, Abcam ab178440, 1:1000; Anti-DsRed, clontech #632496, 1:1000; 6E10, Biolegend #803001, 1:1000; anti-LAMP1, DSHB 1D4B, 1:100) were added and samples were incubated overnight at 4 °C. The samples were washed three times with PBS-T and subsequently incubated with the indicated secondary antibodies (Alexa Flour 488 (A32723), Alexa flour 594 (A11036), Invitrogen, 1:200) for 4 h at 4 °C. The tissues were washed three times with PBS-T and mounted in slow fade gold buffer (Invitrogen).

Immunostaining of adult brains were performed as previously described [36]. Briefly, we dissected the brain tissues of adult flies and fixed them on ice for 30–45 min in fixing buffer (940 µl of 1% PBS-T and 60 µl of 37% formaldehyde). The tissues were washed three times in 0.1% PBS-T and blocked overnight at 4 °C in blocking buffer (1 ml 1 × PBS, 0.1% Triton-X, 5 mg/ml BSA). The tissues were incubated for 16 h at 4 °C with the indicated primary antibodies (anti-TH, Pel-Freez P40101-150, 1:1000; anti-mcherry Abcam ab167453, 1:1000; anti-Ubiquitin, Abcam ab140601, 1:1000; 6E10, Biolegend #803,001, 1:1000; anti-Rab5, Abcam ab31261, 1:1000). Samples were washed three times with 0.1% PBS-T and subsequently incubated with the appropriate secondary antibodies (Alexa Flour 488 (A32723), Alexa flour 594 (A11036), Invitrogen, 1:200) for 4 h at 4 °C. Samples were mounted in slow fade gold buffer (Invitrogen) and viewed using a Leica SP8 confocal microscope.

### Molecular cloning

The Flag-C99 plasmid was generated by inserting C99 coding sequence with stop codon to the pCMV-Flag-Myc vector via the *NotI/BamHI* cloning sites. The following PCR primers were used to amplify C99 sequence from pCAX-C99:

C99-NotI-F TCAGCGGCCGCGGATGCAGAATTCCGACATG.

C99-BamHI-R TCAGGATCCCTAATTCTGCATCTGCTCAAAG.

No ER Flag-C99-myc was generated by deletion of the TGA stop codon from Flag-C99 using the following PCR primers:

Forward GGATCCGAACAAAAACTC.

Reverse ATTCTGCATCTGCTCAAAG.

ER-Flag-C99 was generated by inserting the ER-target sequence of human APP upstream and in-frame with Flag-C99 using the following PCR primers:

Forward CTGGCCGCCTGGACGGCTCGGGCGGACTACAAAGACCATGAC.

Reverse CAGGAGCAGTGCCAAACCGGGCAGCATGGTTAATTCTGACGG.

ER-Flag-C99-myc was generated by deletion of the TGA stop codon from ER-Flag-C99 using the following PCR primers:

Forward GGATCCGAACAAAAACTC.

Reverse ATTCTGCATCTGCTCAAAG.

ER-C99 was generated by deleting the Flag sequence from ER-Flag-C99 using the following PCR primers:

Forward CTTGCGGCCGCGGATGCAG.

Reverse CGCCCGAGCCGTCCAGGC.

ER-C99-VVE-HA was generated by inserting the HA epitope into ER-C99 using the following PCR primers:

Forward TCCAGATTACGCTGTTGACGCCGCTGTCACC.

Reverse ACATCGTATGGGTACTCCACCACACCATGATGAATG.

ER-C99-SK-HA was generated by inserting the HA epitope into ER-C99 using the following PCR primers:

Forward TCCAGATTACGCTATGCAGCAGAACGGCTAC.

Reverse ACATCGTATGGGTACTTGGACAGGTGGCGCTC.

ER-C99-YK-HA was generated by inserting the HA epitope into ER-C99 using the following PCR primers:

Forward TCCAGATTACGCTTTCTTTGAGCAGATGCAG.

Reverse ACATCGTATGGGTACTTGTAGGTTGGATTTTCG.

Ifn-C99 was generated by substituting the human APP ER-targeting sequence in ER-C99 with the signal sequence of Ifn using the following PCR primers:

Forward TTTCAGCTTTGCGTGACTTTGTGTGATGCAGAATTCCGACATG.

Reverse AGCTAAGAAATAAGTTGTATAACTCATGGTTAATTCTGACGG.

Opn-C99 was generated by substituting the human APP ER-targeting sequence in ER-C99 with the signal sequence of Opn using the following PCR primers:

Forward CCTGTTCGGCCTTGCCTCCTGTGATGCAGAATTCCGACATG.

Reverse CAAAAGCAAACCACTGCCAGTCTCATGGTTAATTCTGACGG.

ER-C99-3 K-A was generated by mutating the three K residues in ER-C99 immediately C-terminal of the TM domain of C99 with three A residues using the following PCR primers:

Forward AGCACAGTACACATCCATTCATCATG.

Reverse GCTGCCAGCATCACCAAGGTGATG.

### Cell lines

HeLa cells and HEK293T cells were purchased from ATCC. HEK293T cells stably transfected with Aβ42-YFP were described before [37]. Cells were cultured under standard tissue culture conditions (1 × DMEM medium, GIBCO, 10% FBS, 5% CO2, 37 °C).

### Protein extraction from cultured cells and western blotting

HeLa cells were transfected with the respective plasmids. Cells were washed with 1X PBS 30 h post transfection and lysed in lysis buffer (50 mM Tris–HCl, 150 mM NaCl, 1% Triton X100, protease inhibitors). The cells were centrifuged at 13,000 rpm for 20 min at 4 °C. Protein analysis was carried out by Bradford method. The supernatant was then mixed with 4 × protein loading buffer and loaded onto either 4–12% bis–tris gels using MES as running buffer or on 16% Tricine gel and immunoblotted onto PVDF membranes. The membranes were blocked with blocking buffer (5% BSA in TBST) and incubated with following primary antibodies (Anti-Flag, Sigma-Aldrich F1804, 1:2000; Anti-GFP, ProteinTech 66,002, 1:1000; Anti-ubiquitin, Abcam ab140601, 1:1000; Anti-Actin, Sigma-Aldrich A2228, 1:500; 6E10, Bio Legend 803,001, 1:1000; Anti-Myc, ProteinTech 16,286, 1:1000; Anti-APP C-term (C1/6.1), ThermoFisher 512,700, 1:500; Anti-Calnexin, ProteinTech 1047–2-AP, 1:1000; Anti-RPL22, ProteinTech 25,002-1-AP, 1:1000; Anti-Sec61B, ProteinTech 15,087-1-AP, 1:2000; Anti-HA, Sigma-Aldrich 12CA5, 1:3000; Anti-eRF1, Cell Signaling 13,916, 1:1000; Anti-eRF3, Cell Signaling 14,980, 1:1000; Anti-DDX19B, ProteinTech 18,285-1-AP, 1:1000; Anti-ZNF598, GeneTex GTX119245, 1:250; Anti-ANKZF1, ProteinTech 20,447-1-AP, 1:1000; Anti-NEMF, ProteinTech 11,840-1-AP, 1:500; Anti-Ltn1, ProteinTech 28,452-1-AP, 1:1000; Anti-RPL7a, ProteinTech 15,340-1-AP, 1:1000; Anti-Rack1, Santa Cruz sc-17754, 1:1000; Anti-PSD95, Abcam ab18258, 1:1000). Goat anti-Rabbit IgG HRP, Santa Cruz sc2004 or Goat anti-Mouse IgG-HRP, Santa Cruz sc2005 antibodies were used for detection at 1:10,000 dilution. For data quantification of western blots, signal intensity was measured and calculated using NIH Image J.

Special steps were taken during SDS PAGE to better resolve the different APP.C99 species. We tried to resolve the gel as much as possible such that the lower protein ladder (10–25) was distinctly separated. This often required running the gel front until it was just at the bottom of the gel. Care was taken not to run the ~ 10 KD protein ladder band out as the C99 lower band was just beyond this molecular weight. The gel was transferred onto PVDF membrane using regular 1 × Transfer buffer. The transfer conditions were 350 mA constant for 45 min for the bis–tris gel or 85 V constant for 1 h.

### Membrane fraction isolation

To isolate a crude membrane fraction containing ER, lysosomes and Golgi, cells were washed with 1 × PBS before incubating with 50 mM Tris–Cl, 150 mM NaCl, 25 mg/ml digitonin for 10 min at 4 °C. The cells were then centrifuged at 2000 rpm for 10 min. The lysate was then incubated with buffer containing 1% Triton X100 to solubilize the membrane structures and incubated at 4 °C for 30 min. This was followed by centrifugation at 7000 rpm for 10 min to pellet the nuclei and supernatant containing membrane protein extract.

### Immunoprecipitation

Cells were washed with 1 × PBS 30 h post transfection and lysed using either lysis buffer or 6 M Urea buffer (6 M urea, 50 mM Tris–HCL, 150 mM NaCl, protease inhibitors) as indicated. 5% of the lysate was kept aside to load as input. The remaining lysate was incubated with the desired antibody at 4 °C overnight. If using urea buffer, the lysate was diluted to 0.5 M urea before incubating with the desired antibody (4 µl of 6E10 (Bio Legend), 2 µl for Sec61B (ProteinTech), and 2 µl for Rpl22 (ProteinTech) antibodies). The following day protein A/G beads (Santa Cruz sc2003) were added to the antibody-lysate mixture and incubated for 2hs at 4 °C. The beads were then washed with lysis buffer three times and the wash was removed by centrifugation. The beads were suspended in 4 × SDS loading buffer and boiled for 5 min before loading onto bis–tris gels.

### Immunostaining of cultured cells

Cells on coverslips were washed with PBS twice and fixed in 4% paraformaldehyde/PBS solution for 15 min at RT. Cells were washed repeatedly with 1 × PBS prior to incubating with PBS containing 0.1% Triton X-100 for 20 min. Cells were then incubated in blocking buffer (1 × PBS, 0.1% Triton X100, 2% BSA) for 30 min. After blocking, desired primary antibodies (Anti-Rab5, Abcam ab31261; Anti-LC3, ProteinTech 14,600-1-AP; 6E10, BioLegend 803,001; Anti-Calnexin, ProteinTech 10,427-2-AP; Anti-Puromycin, Millipore MABE343; M78, Gift from Dr. Glabe) were added to the blocking buffer at 1:1000 concentration and cells were incubated with the antibody solution overnight. After washing with 1 × PBS the following day, cells were incubated in appropriate secondary antibodies for 1 h. Cells were washed again and the coverslips were mounted on slides using DAPI-containing mounting medium.

### MTT assay

HeLa cells were plated one day before transfection with plasmids and siRNAs as noted in the text. The medium was changed after overnight transfection. 30 h post transfections, the DMEM containing 0.5 mg/ml MTT reagent (Sigma M2128) was added to the cells. The cells were incubated at 37 °C for 120 min, and the medium was removed following which the purple formazan crystals were dissolved using DMSO. After shaking on a shaker for 30 min to ensure complete dissolution of the crystals, the resulting absorbance was measured at 570 nm.

### Plasmid transfections and siRNA knockdown

Cell transfections were performed by using Lipofectamine 3000 (cat#: L3000015, Invitrogen), and siRNA knockdown experiments were performed using Lipofectamine RNAiMAX reagent (cat#: 13,778,150, Invitrogen), according to manufacturer’s instructions. Different plasmids were used for transfection of HeLa and HEK293T cells using lipofectamine 3000 following instructions from the manufacturer. Briefly, cells were plated at 70% confluency the day before in DMEM without antibiotics. On the day of transfection, plasmid DNA and lipofectamine reagent were individually mixed in OptiMEM and then mixed together such that the plasmid/reagent = 1:3. After incubation of 10 min at room temperature, the plasmid-reagent mixture was added dropwise to the cells. Medium was changed the following day and cells were analyzed 30 h post transfection. For siRNA treatments, a similar protocol was followed with the final concentration of siRNA at 450 pmol for a 10 cm dish (for a 10 cm dish: 10 µg of DNA and 20 µl of Lipofectamine 3000, for 6 cm dish: 3–5 µg of DNA and 5 µl of Lipofectamine 3000 were added). For siRNA and plasmid co-transfection, the cells were first treated with siRNA. 24 h post transfection with siRNA, the medium was replaced and plasmid DNA transfection was carried out.

### Translocon capture with ConA beads

Proteins were extracted from cells grown on 10 cm culture dishes. Proteins were extracted as indicated either in Urea buffer or crude membrane preparations. This was followed by incubating for 2hs at 4 °C with 50 µl of Con A beads (Sigma-Aldrich GE17-0440–03). The beads were washed with lysis buffer three times before adding the 4X SDS loading buffer. For RNAse A and EDTA treatments, membrane fractions were isolated from HeLa cells transfected with APP.C99 as described before followed by capture with ConA beads. After washing the beads as described before, the beads were centrifuged and RNaseA (1 µg/ml) and EDTA (50 mM) were added to the beads. RNAse A treatment was incubated at 37 °C for 30 min while EDTA treatment was left on ice for 30 min. An aliquot of untreated beads was included as control. After the treatment period, the beads were again centrifuged. Supernatants and beads were then separated and mixed with SDS loading buffer, boiled and loaded onto 4–12% Bis–Tris gel.

### Puromycin labeling of ribosome stalled newly synthesized proteins

At ~ 30 h post transfection, HeLa cells were incubated with fresh DMEM medium containing HHT (5 µM) for 10 min at 37 °C. Emetine (100 µM) and Puromycin (100 µM) were then added to the medium and cells were incubated further for 15 min at 37 °C. Cells were then washed and harvested in lysis buffer, following which proteins were extracted as previously described.

Puromycin labeling of stalled proteins: Puromycin labeling was done as described [38], with slight modifications. Hela cells were seeded on coverslips in a 6-well plate and transfected with pCAX-C99 (Addgene #30,146). After 48 h transfection, cells were treated with HHT for 5 min, thereafter Puromycin (50 µg) and Emetine (100 µg) were added and cells were incubated for 5–7 min. Once the incubation was over, cells were permeabilized by 0.02% digitonin in Permeabilization buffer (50 mM Tris–HCl, pH7.5, 5 mM MgCl2, 25 mM KCl, 355 mM cyclohexamide, 10 units RNAseOut and 0.02% digitonin) for 2 min. Permeabilized cells were washed twice with washing buffer (permeabilization buffer without digitonin) and fixed in 4% paraformaldehyde for 30 min. The Permeabilization and washing steps were performed in ice-old buffers. Cells were then stained with the mOC78 and Puromycin antibody and observed under the confocal microscope.

### Drug treatments

HeLa cells were treated with the following drug concentration and times as indicated in the main text. Cycloheximide: 50 µg/ml for 4 h; HHT: 5 µM for 10 min; emetine: 100 µM for 15 min; Puromycin: 100 µM for 15 min.

### Aβ42-YFP aggregation assays

Aβ42-YFP cells were described before [37]. The pCAX-C99 (Addgene #30,146) plasmid was transfected into Aβ42-YFP cells using Lipofectamine 3000 according to the Manufacturer’s manual. 48 h after transfection, YFP signal was detected under confocal microscope. For siRNA knockdown experiments, Aβ42-YFP cells at 50% confluence were transfected with siRNA targeting ANKZF1 (Invitrogen HSS113541), ABCE1 (Invitrogen HSS109285), VCP (Invitrogen HSS123962), NEMF (Invitrogen HSS113541) and ZNF598 (Invitrogen HSS132049) for 24 h using RNAiMAX (Invitrogen #13778150), according to the manufacturer's instructions. Then cells were transfected with pCAX-C99 plasmid for further 48 h. The cells were washed with 1 × PBS and fixed with 4% paraformaldehyde for 15 min. YFP signals were acquired using confocal microscope (Leica, SP8).

For immunostaining assays, Aβ42-YFP cells were transfected with ER-FLAG-C99 and FLAG-C99 respectively for 48 h. Cells were fixed with 4% paraformaldehyde for 15 min and permeabilized with 0.5% Triton X-100 for 15 min. After blocking with 5% BSA for 1 h, cells were incubated with anti-FLAG antibody (Sigma-Aldrich, F7425) at room temperature for 2 h. Slides were washed three times with PBS and incubated with Alexa Fluor 633-conjugated secondary antibodies for 1 h at room temperature. Images were acquired using confocal microscope (Leica, SP8).

### Mouse studies

B6SJL-Tg(APPSwFlLon,PSEN1*M146L*L286V)6799Vas/Mmjax mice (5xFAD mice) were purchased from Jackson Laboratory. The mice were bred and housed in the Stanford University animal facility. All mouse experiment-related protocols were approved by the Stanford’s University School of Medicine’s APLAC committee.

Lenti-scramble control shRNA viral particles expressing GFP (lenti-GFP) and mouse ZNF598 shRNA lentiviral particles expressing GFP (Lenti-shZNF598) were purchased from OriGene (CAT#: TL513709V). Viral titers were > 10E7 TU/ml. Neonatal pups were injected with either Lenti-GFP or Lenti-shZNF598 at P0/P1 stage as described before [39]. Briefly, pups were cryo-anaesthetized before injection. Following cessation of any movement, the pups were placed side up and 2 µl of virus was injected on each side with a 32 gauge needle (BD catalogue #324,909). The injection site is about 2/5th of the distance between the eye and the lambda intersection as described before [39]. Following injection, the pups were warmed and returned to the cage with the mother for further care. Mice were sacrificed after 5 months. Brain samples were dissected and subjected to fixation with 4% PFA and later treatment with 10% and 20% sucrose for 24 h. Frozen sectioning was performed according to Stanford Pathology facility protocols. Subsequent immunostaining was carried out by following standard procedures.

### Synaptosome isolation from mouse brain

Mouse brain tissue was homogenized in cold lysis buffer (0.32 M sucrose, 5 mM HEPES, complete protease and phosphatase inhibitors) at 5:1 buffer to tissue (mg) volume using a mechanical homogenizer for 30 s at 800 rpm. The resulting homogenate was centrifuged at 1000 g at 4 °C for 10 min to separate the nuclei pellet. The supernatant was further centrifuged at 12000 g for 10 min at 4 °C to pellet the synaptosomes. The pellet was further suspended in 1 × TBS solution and used for SDS gel electrophoresis. The purity of the synaptosomal isolation was checked by detection of PSD95 protein in the pellet but not supernatant fraction.

### Mouse brain frozen section immunofluorescence staining

Animals were sacrificed by cervical dislocation and subsequently harvested followed by storing in 4% formalin (Sigma) for 24 h. Tissues were then moved into 10% sucrose solution for 24 h and 20% sucrose for 24 h sequentially. After processing, tissues were embedded with OCT. Ten-micrometer sections were fixed in cold acetone for 10 min and then air dried at room temperature for 1 h. In order to remove OCT, slides were placed in 1 × PBS wash buffer for 10 min followed by processing with 0.3% PBST. After washing with 1xPBS, excess wash buffer was wiped off from slide without drying sections. Tissues were blocked with 5% goat serum for 1 h at room temperature followed by incubating with primary antibodies (Anti-IBA1, Novus NBP2-19,019; Anti-ZNF598, Genetex GTX119245; Anti-Rack1, Santa Cruz sc-17754; MOAB2, Novus NBP2-13,075; Anti-CTSD, ProteinTech 55,021-1-AP; Anti-Lamp1, DSHB 1D4B; Anti-RPL22, ProteinTech 25,002–1-AP) overnight at 4 °C. Samples were rinsed in wash buffer for 5 min. After wiping off excess buffer, samples were incubated with secondary antibodies (conjugated to fluorophores) in the dark for 2 h at room temperature. After washing with PBS for 3 times, samples were ready for mounting with mounting media. Images were taken on a Leica SP8 confocal microscope.

Immunostaining for Cathepsin D (Abcam), 6E10 (Bio Legend), Rab5 (Abcam), LAMP1 (DSHB), P62 (Abcam), and IBA-1 (Novus) were performed on frozen brain tissue sections (5 μm). Briefly, the frozen sections were washed in 1 × PBS, and then permeabilized with 0.5% Triton X-100 for 15 min, blocked with 3% goat serum. Slides were incubated with anti-Cathepsin D, 6E10, Rab5, LAMP1, P62, IBA-1 antibody overnight at 4 °C. Slides were washed three times with PBS and incubated with Alexa Fluor 488- and 569-conjugated secondary antibodies for 1 h at room temperature. Slides were washed three times with PBS and mounted.  Images were acquired using confocal microscope (Leica, SP8). The number of positive dots labeled by Cathepsin D or LAMP1 were counted in each cell.

### Human subjects

Human tissue samples from patients with AD and controls were provided by the UCSF Neurodegenerative Disease Brain Bank. Demographic information is provided in Supplementary Table 1. Informed consent to undergo autopsy was provided by patients and/or their surrogates, following the principles outlined in the Declaration of Helsinki. Formalin-fixed, paraffin-embedded tissue blocks from the inferior temporal gyrus and/or middle frontal gyrus were cut into 8-microns thick sections and mounted on glass slides.

### Paraffin section immunofluorescence staining of human brain samples

AD and control human brain sections were deparaffinized in xylene and rehydrated through graded ethanol series. Antigen retrieval was performed by incubating the slides in a steamer with citrate buffer (pH 6.0) (Sigma) for 30 min. The slides were then washed for 10 min in 1 × PBS and incubated in 3% H_2_O_2_ (in methanol) for 10 min in order to block any endogenous peroxidase activity. Slides were then washed with 1 × PBS for 10 min. To prevent nonspecific binding and excessive background, slides were blocked with a serum-free buffer for 30 min. Primary antibodies (Anti-Rack1, Santa Cruz sc-17754, 1:500; Anti-ZNF598, GeneTex GRX119245, 1:250; Anti-ABCE1, Gift from Dr. Hegde; Anti-NEMF, ProteinTech 11,840-AP, 1:500; Anti-RPL22, ProteinTech 25,002-1-AP, 1:1000; Anti-ANKZF1, ProteinTech 20,447–1-AP, 1:1000) were applied on the slides and incubated overnight at 4 °C. Slides were washed with PBS and incubated with secondary antibody (conjugated to fluorophores) for 2 h at room temperature. After washing with 1 × PBS, mounting medium was used to mount the slides. Images were taken by Leica SP8 microscope.

### Quantification and statistical analysis

Statistical analysis was performed using GraphPad Prism 8 (Windows version 8, GraphPad Software, San Diego, CA, USA). Student’s t test and one-way ANOVA test with Tukey’s post hoc test were used for statistical evaluation. All data are represented as mean ± S.D, with P < 0.05 being considered statistically significant. ^∗^*p* < 0.05, ^∗∗^*p* < 0.01, ^∗∗∗^*p* < 0.001.

## Results

### APP.C99-induced neuromuscular defects in *Drosophila*

**Fig. 1:**
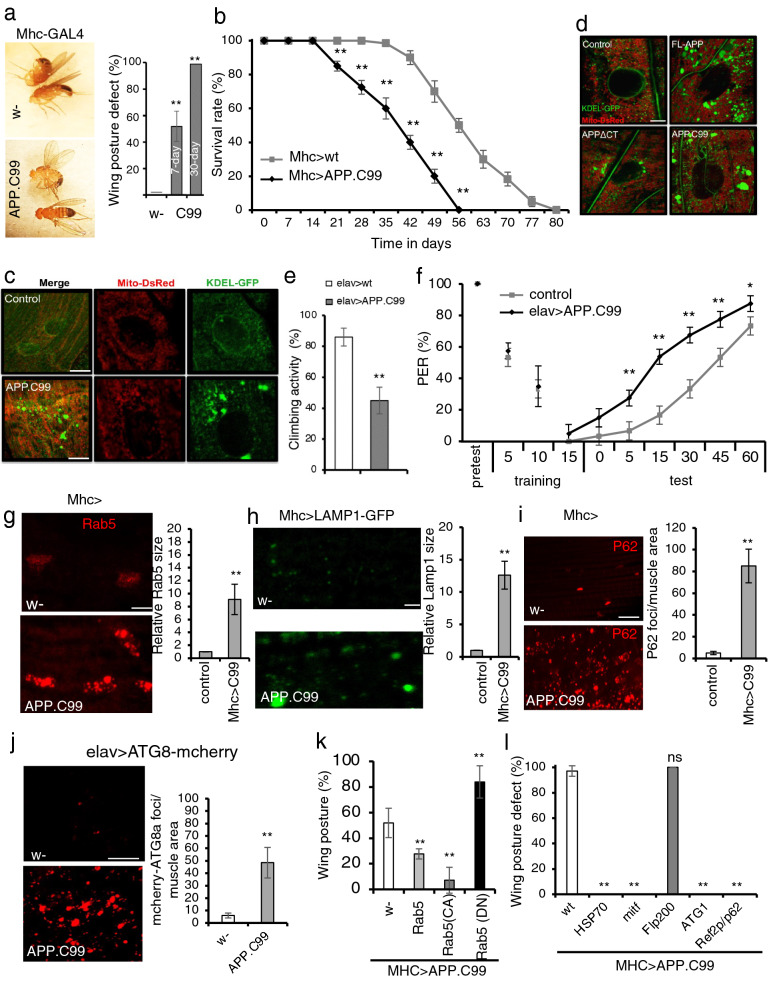
Endolysosomal and autophagy defects contribute to the neuromuscular toxicity of APP.C99 in *Drosophila.* **a** Images showing effect of muscle expression of APP.C99 on wing posture. Bar graph shows penetrance of wing posture defects at different ages (n > 100). **b** Effect of muscle expression of APP.C99 on fly lifespan (n = 80). **c** Immunofluorescent images showing effect of muscle expression of APP.C99 on ER (KDEL-GFP) and mitochondrial (mito-DsRed) morphology at the larval body wall muscle. Scale bar, 10 µm. **d** Images comparing effects of FL-APP, APPΔCT, and APP.C99 on ER and mitochondrial morphology. Scale bar, 5 µm. **e** Effect of neuronal expression of APP.C99 on locomotor activity (n = 80–100). **f** Effect of neuronal APP.C99 expression on learning and memory in the aversive taste memory assay (n = 40–60). **g-i** Immunostaining and data quantification showing accumulation of enlarged early endosomes as detected with anti-Rab5 antibody (**g**), enlarged lysosomes as detected with LAMP1-GFP reporter (**h**), and the autophagy receptor p62 (**i**) in fly muscle expressing APP.C99 (n = 10). Scale bars, 5 µm (**h**), 10 µm (**g, i**). **j** Accumulation of autophagosome reporter ATG8-mcherry in fly brain expressing APP.C99 (n = 10). Scale bar, 20 µm. **k** Effect of co-expression of Rab5-WT, -CA, -DN on APP.C99 induced wing posture defect (n = 80). **l** Effect of co-expression of Hsp70, Mitf, Fip200, ATG1, and Ref2p/p62 on APP.C99 induced wing posture defect (n = 80). Error bars, ± SEM; ^∗^ *P* < 0.05, ** *P* < 0.01 in Student’s *t*-tests

APP.C99 is known to cause neuromuscular toxicity in mammalian systems [40, 41], and recent studies provided compelling evidence that familial *APP* and *PSEN* mutations have discordant effects on Aβ production but similar effects on APP.C99, which accumulates in mutant neurons and exert Aβ-independent toxicity [42–47]. To study mechanism of APP.C99 pathogenesis, we used *Drosophila* as a model system, which has been instrumental for studying APP biology relevant to AD [48–50]. We used a transgene expressing the C99 fragment of human APP protein retaining the N-terminal ER-targeting signal of APP and with a Myc tag at the C-terminus. The transgene was tissue-specifically expressed using the binary UAS-Gal4 system. Flies expressing APP.C99 in the muscle under *Mhc-Gal4* control (*Mhc* > *APP.C99*) showed held-up or droopy wing posture (Fig. 1a), an indication of indirect flight muscle degeneration [35]. These flies also exhibited shortened lifespan (Fig. 1b). This result of C99 toxicity in fly muscle resonates with previous studies of a C99 Tg mouse model, which exhibits inclusion body myositis (IBM)-like muscle lesions [51]. We next examined the effect of APP.C99 on muscle cell homeostasis by monitoring mitochondrial and ER morphology using genetically encoded reporters mito-DsRed and KDEL-GFP. Mitochondria were more fragmented in *Mhc* > *APP.C99* than control flies (Fig. 1c). Strikingly, the ER exhibited more drastic morphological alterations, with large aggregated ER structures forming in *Mhc* > *APP.C99* flies (Fig. 1c). Importantly, full-length APP (FL-APP) had similar effect as APP.C99 on ER morphology, whereas deletion of the APP.C99 region from FL-APP significantly attenuated such effect (Fig. 1d), supporting that the phenotypes seen with the APP.C99 transgene reflect the effect of APP.C99 in the FL-APP context. As the aggregated ER structures tended to be in contact with mitochondria, and ER-mitochondria contacts are involved in Ca^2+^ transfer from ER to mitochondria [52], we used a mito-GCaMP reporter to monitor mito-Ca^2+^ and observed increased mito-Ca^2+^ in *Mhc* > *APP.C99* fly muscle (Additional file 1: Fig. S1a), suggesting that APP.C99 affects ER-mitochondrial Ca^2+^ signaling.

We next tested the neuronal effect of APP.C99 using the pan-neuronal *elav-Gal4* driver. These flies exhibited reduced locomotor activity (Fig. 1e). Using the larval neuromuscular junction as a model system for synapse development and morphogenesis, we found that APP.C99 caused reduced expression of the postsynaptic marker Dlg, the fly counterpart of PSD95 (Additional file 1: Fig. S1b), and synapse loss. To correlate the synaptic defects with animal behavior, we next tested the effect of APP.C99 on learning and memory. We measured the aversive taste memory, which is controlled by a neural circuit involving dopaminergic input to neurons in the mushroom body, the memory center of the flies, and downstream mushroom body output neurons that alter taste response [34]. Applying sucrose solution (appetitive tastant) to the tarsi (feet) of a starved, tethered fly induced robust feeding behavior as measured by the proboscis extension reflex (PER). Repeated paired application of sucrose to the tarsi and quinine (aversive tastant) to the proboscis resulted in significant attenuation of the PER response to subsequent application of sucrose alone. Pan-neuronal expression of APP.C99 caused significant impairment of this taste memory (Fig. 1f). Similar impairment of taste memory was also observed in flies producing APP.C99 by co-expressing FL-APP and β-secretase (BACE1) (Additional file 1: Fig. S1c). Thus, neuronal APP.C99 can cause memory deficit. Immunostaining of wholemount brain samples showed wide-spread accumulation of ubiquitin (Ub)-positive aggregates, indicating proteostasis failure (Additional file 1: Fig. S1d). To examine the effect of APP.C99 on the maintenance of specific neuronal types, we chose the DA neurons that can be identified by immunostaining with the anti-tyrosine hydroxylase (TH) antibody. APP.C99 caused a reduction of DA neuron number in the PPL cluster (Additional file 1: Fig. S1e). This was correlated with increased mito-Ca^2+^ level in APP.C99-expressing DA neurons (Additional file 1: Fig. S1f). These data indicate that the fly APP.C99 model recapitulates many key features of AD.

### APP.C99-induced endolysosomal and autophagy defects

Recent studies indicate that endolysosomal and autophagy defects may represent some of the earliest pathogenic events in AD [53]. We found that in APP.C99-expressing fly muscle (Fig. 1g) or brain (Additional file 1: Fig. S1g), there was enlargement of Rab5-positive early endosomes and accumulation of enlarged LAMP1-positive lysosomes (Fig. 1h). There was also increased p62-positive and ATG8/LC3-positive structures, indicating autophagic flux defects (Fig. 1i, j). Consistent with this notion, APP.C99-derived 6E10-positive aggregates were found in perinuclear endolysosomal compartments (Additional file 1: Fig. S1h). APP.C99-induced early endosomal and autophagic flux defects were also observed in mammalian cells (Additional file 1: Fig. S1i, j), in which APP.C99 had similar effect as bafilomycin treatment, which blocks autophagic flux, on p62 and LC3 protein levels, and it specifically altered lipidated LC3II level by increasing LC3II/LC3I ratio (Additional file 1: Fig. S1k).

Whether the endolysosomal and autophagic defects observed in AD are the cause or consequence of the primary drivers of the disease process remains controversial [54]. We next tested the contribution of the endolysosomal and autophagy systems to APP.C99 pathogenesis. Overexpression of a constitutively active Rab5 (Rab5-CA) fully rescued APP.C99-induced muscle toxicity as measured with the wing posture assay, whereas a dominant-negative Rab5 (Rab5-DN) had opposite effect (Fig. 1k). Enhancing autophagy activity by overexpression of Ref2p/p62, an autophagy receptor, ATG1, a kinase required for the initial formation of autophagosomes, or MITF, a transcription factor that regulates the expression of autophagy and lysosome-related genes, also fully rescued C99 toxicity in the fly muscle (Fig. 1l). Overexpression of the molecular chaperone Hsp70A1L had similar effect (Fig. 1l), supporting the importance of proteostasis failure in APP.C99 pathogenesis. Notably, overexpression of the autophagy regulator Fip200 had no obvious effect (Fig. 1l), which not only served as a specificity control of the aforementioned genetic interactions but also indicated that APP.C99 might affect specific steps of the autophagy process. Importantly, the abnormal wing posture caused by FL-APP was also rescued by genetic manipulation of the endolysosomal and autophagy systems and Hsp70A1L (Additional file 1: Fig. S1k), suggesting that endolysosomal and autophagy defects and proteostasis failure also underlie APP.C99 toxicity in FL-APP context.

### ER-associated stalled translation of APP.C99

**Fig. 2:**
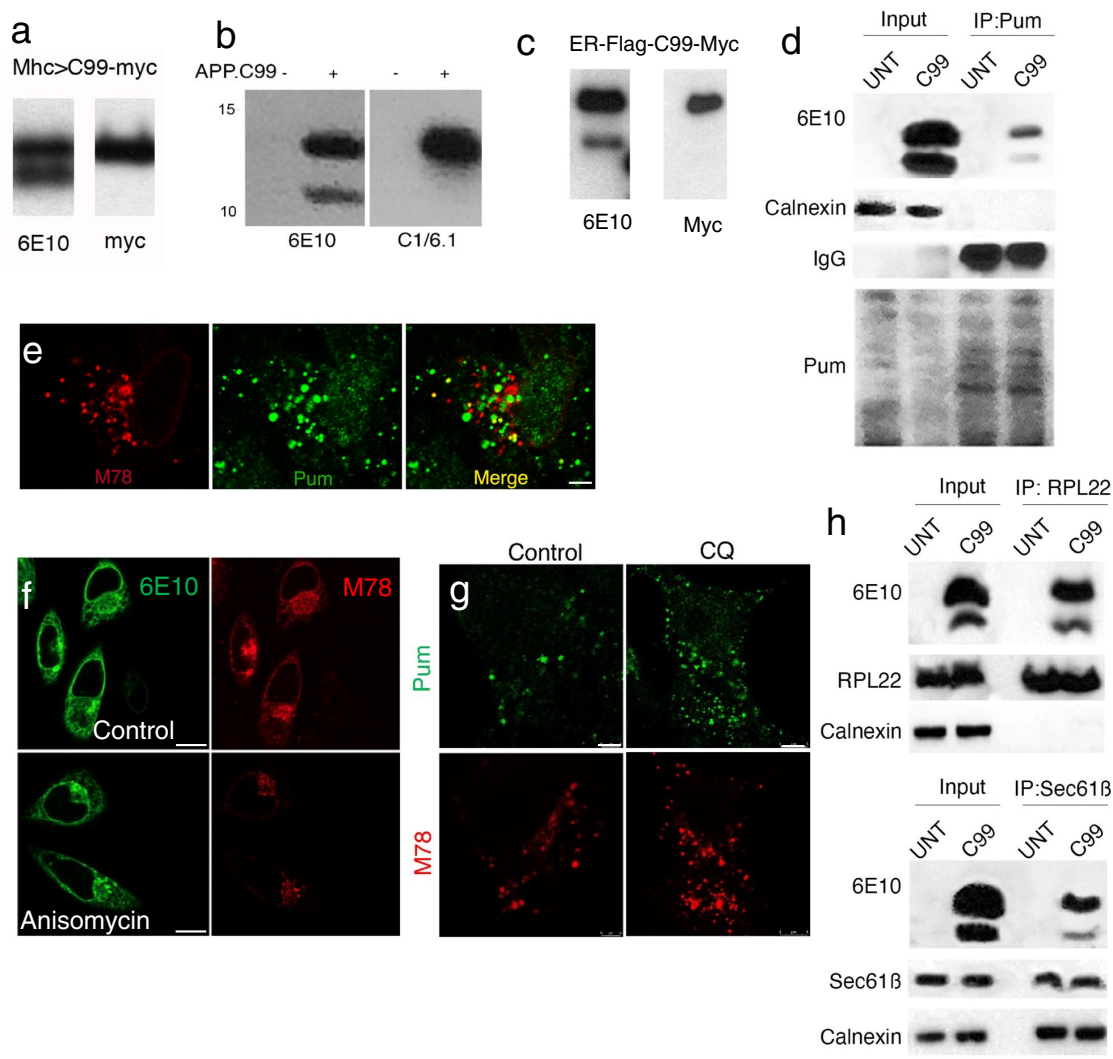
Ribosome stalling during co-translational ER translocation of APP.C99. **a** WB analysis of APP.C99 proteins from fly muscle expressing an APP.C99 transgene with a Myc tag at the C-terminus, using the N-term 6E10 and C-term Myc antibodies. **b** WB analysis of APP.C99 proteins from HeLa cell expressing APP.C99, using the N-term 6E10 and C-term C1/6.1 antibodies. **c** WB analysis of APP.C99 proteins from HeLa cell expressing ER-Flag-C99-Myc, using the N-term 6E10 and C-term Myc antibodies. **d** Pum labeling of stalled APP.C99 NPCs. Stalled NPCs were labelled with emetine plus Pum after HHT treatment to let actively translating ribosomes to run off. Pum-labelled protein were immunoprecipitated with anti-Pum and the presence of APP.C99 was detected with 6E10. The ER protein Calnexin serves as negative control. **e** Immunostaining of APP.C99 transfected HeLa cells with the amyloid conformation-specific mOC78 antibody and anti-Pum, which labeled stalled NPCs after active ribosomes were let run off by HHT treatment. Scale bar, 5 µm. **f** Immunostaining of APP.C99 transfected HeLa cells with 6E10 and mOC78 antibodies in control and anisomycin treatment conditions. Scale bar, 10 µm. **g** Immunostaining of APP.C99 transfected HeLa cells with mOC78 and Pum antibodies in control and anisomycin treatment conditions. Scale bar, 5 µm. **h** Co-IP assay in untransfected (UNT) and APP.C99 transfected HeLa cells. Lysates were immunoprecipitated with anti-RPL22 or anti-Sec61B antibodies, and the immunocomplexes were probed with the indicated antibodies. Immunoblots represent at least 3 independent repeats

Western blot analysis showed that the APP.C99 transgene produced two major protein bands in the fly neuromuscular tissues. The upper band represented full-length APP.C99 as it was immunoreactive to antibodies against the very N-terminal (6E10) and C-terminal (Myc tag) epitopes, whereas the lower band was only reactive to the N-term (6E10) antibody (Fig. 2a). To elucidate the molecular nature of the different APP.C99 species, we resorted to mammalian cell culture studies. HeLa cells exhibited C99-dependent APP toxicity (Additional file 1: Fig. S2a), and signal sequence-dependent APP.C99 toxicity (Additional file 1: Fig. S2b), and APP.C99 was localized perinuclearly and enriched in endolysosomal compartments (Additional file 1: Fig. S2c), suggesting that these cells can be used to study mechanisms of APP.C99-induced cytotoxicity.

As in fly neuromuscular tissues, HeLa cells expressing APP.C99 targeted to the ER with the signal sequence of human APP produced the full-length APP.C99 (FL-APP.C99), but also a lower molecular weight (MW) band, which was reactive with the 6E10 antibody recognizing the N-terminus of APP.C99, but not the C1/6.1 antibody recognizing the very C-terminus of APP.C99 (Fig. 2b). This lower band is not the C83 fragment resulting from α-secretase cleavage, which would be recognized by the C1/6.1 but not 6E10 antibody. Similarly, WB analysis of an ER-targeted C99 with a C-term Myc tag detected two bands with the 6E10 antibody but a single band with anti-Myc, which corresponded to FL-APP.C99 (Fig. 2c).

The lower band could be a processed form of FL-APP.C99, or an arrested translation product resulting from ribosome stalling. To distinguish between these possibilities, we performed puromycin (Pum) labeling of stalled NPCs. It involved a pre-treatment of cells with homoharringtonine (HHT), which blocks formation of the first peptide bond but allows actively elongating ribosomes to run off [38]. This was followed by a combined emetine and Pum treatment to incorporate the tRNA-like Pum to the very C-termini of ribosome-stalled NPCs to terminate translation. The translation elongation inhibitor emetine is needed for Pum to be efficiently incorporated into stalled NPCs [38]. After IP with anti-Pum to pull down stalled NPCs, followed by probing with 6E10, we found that both APP.C99 species were Pum-labeled, supporting that each contained stalled NPCs (Fig. 2d). The fact that the lower band has the very N-term 6E10 epitope and C-term Pum argues against it being any kind of processed product. Immunostaining with anti-Pum antibody showed that stalled NPCs were localized to the perinuclear ER compartment where some of them colocalized with the conformation-specific mOC78 antibody (Fig. 2e), which recognizes a discontinuous epitope consisting of residues 8–12 (SGYEV) and 24–26 (VGS) in misfolded or aggregated Aβ [55, 56], suggesting that stalled NPCs adopted abnormal conformation and represented aberrant APP.C99 species. Thus, the translation of APP.C99 is stalled at two sites: one at or near the stop codon, another at an internal site ~ 30 AA upstream of the stop codon based on the size of the lower band. Interestingly, treatment of APP.C99 transfected HeLa cells with the translation inhibitor anisomycin (Fig. 2f) significantly reduced the perinuclear mOC78-positive signals, whereas the signals in the endolysosomal compartment was less affected, suggesting that the stalled NPCs were synthesized at the perinuclear ER membrane and later entered the endolysosomal compartment. Treatment with the lysosomal inhibitor chloroquine resulted in a robust increase of mOC78-positive stalled NPCs (Fig. 2g), suggesting that the lysosome is actively involved in the turnover of aberrant APP.C99 species.

One prediction of the translational stalling model is that both APP.C99 species would exhibit prolonged interaction with the ribosome and the ER-translocon. Indeed, both APP.C99 species were present in ribosomes and we detected their co-IP with 60S ribosome (RPL22) and translocon (Sec61B) proteins (Fig. 2h). We also used concanavalin A (ConA) immobilized on agarose beads to enrich for ER translocon and associated proteins [57], and found that both APP.C99 species associated with ER translocon, and that such associations were sensitive to RNase A or EDTA treatment (Additional file 1: Fig. S2d), indicating ribosome/mRNA-ribonucleoprotein (mRNP)-mediated translocon engagement.

To estimate the approximate location of the internal stalling site, we made APP.C99 constructs in which the HA tag was inserted at various positions after the transmembrane (TM) domain (Additional file 1: Fig. S2e). Western blot analysis showed that the HA inserted at the VVE site was present in both APP.C99 species, whereas the HA inserted at the SK or YK sites were present in FL-APP.C99 but not the internally stalled species (Additional file 1: Fig. S2f). Moreover, the internally stalled species from the VVE insertion was slightly larger than that from the SK and YK insertions, suggesting that the internal stalling site is located after the VVE but before the SK insertion sites.

### ABCE1 deficit contributes to APP.C99 ribosome stalling at the stop codon site

**Fig. 3:**
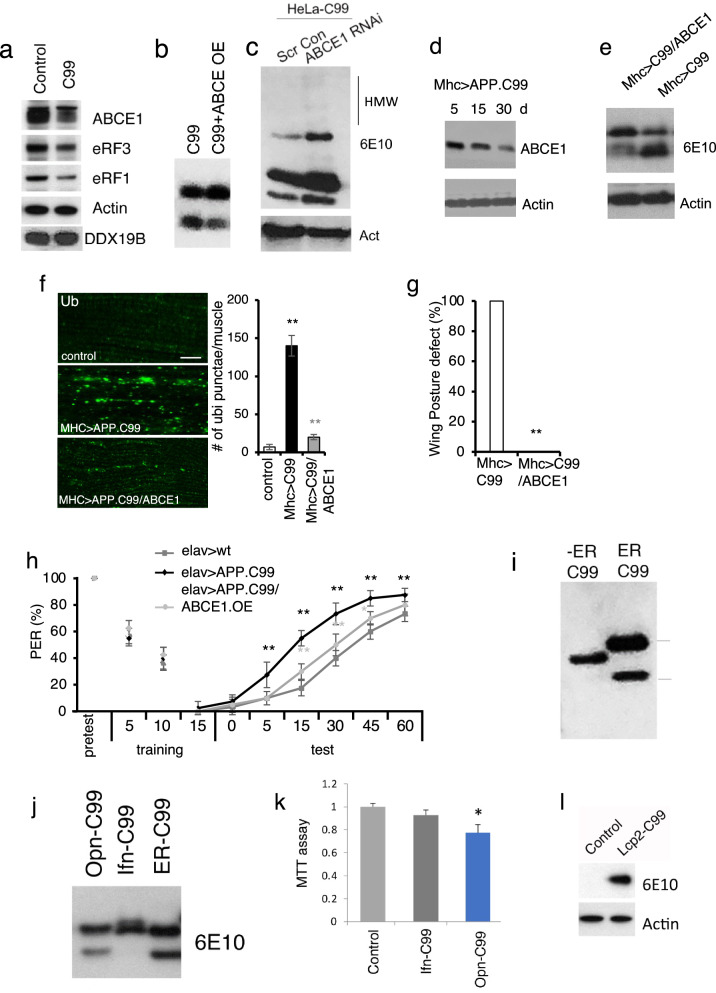
ER targeting and ABCE1 deficiency contribute to stalled translation of APP.C99. **a** Immunoblots showing altered levels of ABCE1 and eRF1/3 in APP.C99 transfected HeLa cells. **b, c** Immunoblots showing effect of ABCE1 OE (**b**) or RNAi (**c**) on the level of stalled APP.C99. **d** Immunoblots showing ABCE1 level in muscle tissue of young and old *Mhc* > *APP.C99* flies. **e** Effect of ABCE1 OE on the level of stalled APP.C99 in fly muscle. **f** Immunostaining and data quantification showing effects of ABCE1 OE on the level of P62-positive protein aggregates in *Mhc* > *APP.C99* fly muscle (n = 10). Scale bar, 10 µm. **g, h** Effect of ABCE1 OE on wing posture defect (**g**) and aversive taste memory (**h**) of *elav* > *APP.C99* flies (n = 80–100). **i** Immunoblots showing the effect of removing signal sequence on the translational stalling of APP.C99. **j** Immunoblots showing the effect of alternative signal sequence on the translational stalling of APP.C99. **k** MTT cell viability assay of HeLa cells transfected with the indicated plasmids. **l** Immunoblots showing the effect of replacing APP signal sequence with that of Lcp2 on the translational stalling of APP.C99 in fly muscle. Error bars, ± SEM; ^∗^*P* < 0.05, ^**^P < 0.01. Immunoblots represent at least 3 independent repeats

Ribosome stalling at native stop codon sites could be due to an insufficient supply of ribosome termination or recycling factors such as ABCE1, eRF1, or eRF3, as previously shown for ribosome stalling caused by mitochondrial stress during C-I30 translation [58], whereas stalling at internal sites could happen for various reasons, e.g., co-translational protein folding, targeting to the ER, or membrane integration and topogenesis [59], all of which could contribute to the slowdown and stalling of APP.C99 translation on the ER membrane. To test the mechanism of ribosome stalling during APP.C99 translation, we examine the level of termination and recycling factors in APP.C99 expressing cells. We found decreased levels of ABCE1, eRF1, and eRF3, whereas DDX19B was not affected (Fig. 3a). Importantly, overexpression of the ribosome recycling factor ABCE1 in HeLa cells reduced the level of stalled APP.C99 as detected by WB (Fig. 3b) and Pum labeling (Additional file 1: Fig. S3a) of stalled NPCs. The amount of APP.C99 localized to the perinuclear ER and endolysosomal compartments (Additional file 1: Fig. S3b) was also greatly reduced. On the other hand, ABCE1 RNAi increased the level of stalled APP.C99 (Additional file 1: Fig. S3a) and it altered autophagic flux as indicated by elevated p62 and LC3 protein levels (Additional file 1: Fig. S3c), although LC3II/LC3I ratio was unexpected decreased. Moreover, some higher MW species of APP.C99, presumably oligomers, were induced by ABCE1 RNAi (Fig. 3c). Thus, inefficient ribosome termination/recycling leads to aberrant APP.C99 products.

We next tested the physiological role of ABCE1 in regulating APP.C99 translational stalling and toxicity in vivo. The wing posture phenotype of *Mhc* > *APP.C99* flies worsened with age (Fig. 1a). This was correlated with an age-dependent reduction of ABCE1 protein level (Fig. 3d), suggesting that loss of ABCE1 protein may contribute to APP.C99 toxicity. Indeed, overexpression (OE) of ABCE1 effectively rescued the translational stalling (Fig. 3e) and impaired proteostasis caused by APP.C99 (Fig. 3f), and the pathological effects of APP.C99 at the whole animal level as measured by the wing posture (Fig. 3g) and aversive taste memory (Fig. 3h) assays. Moreover, ABCE1 OE rescued the synaptic defect caused by FL-APP (Additional file 1: Fig. S3d) and the memory defect of *APP/BACE1* flies co-expressing FL-APP and BACE1 (Additional file 1: Fig. S3e). These data suggest that the ABCE1 deficit is a key contributor of stalled APP.C99 translation and ensuing toxicity, and that the modifying effect of ABCE1 on APP.C99 toxicity is relevant in the FL-APP context.

### ER-targeting and membrane topogenesis contribute to ribosome stalling at the APP.C99 internal stalling site

We next examined the mechanism of ribosome stalling at the internal site. Strikingly, removal of the ER-targeting signal, which abolished APP.C99 toxicity as shown earlier (Additional file 1: Fig. S2b), prevented the formation of the APP.C99 lower band (Fig. 3i) and the mOC78-positive aberrant protein species (Additional file 1: Fig. S3f). Despite repeated attempts, AICD, the product of γ-secretase cleavage product of APP.C99, was undetectable in ER-C99 or No-ER-C99 transfected HeLa cells. Thus the targeting of APP.C99 to the ER is an important event leading to ribosome stalling and generation of aberrant APP.C99 species, which is the main cause of APP.C99 toxicity. Interestingly, replacing the ER-targeting signal of APP with that of IFN-γ, which is known to be competent for ER targeting but inefficient in engaging the Sec61 translocon [60], prevented the internal stalling (Fig. 3j). On the other hand, the signal sequence of Osteopontin (OPN), which engages the translocon efficiently [60], was as effective as the native APP signal sequence in conferring APP.C99 internal stalling (Fig. 3j). Consistent with internal stalling being cytotoxic, OPN-C99 but not IFN-γ-C99 impaired cell viability (Fig. 3k). Moreover, replacing the APP signal sequence with that of fly larval cuticle protein 2 (Lcp2), a secreted protein, greatly reduced the internal translational stalling of C99 (Fig. 3l), as in the case of IFN-γ-C99 in mammalian cells (Fig. 3j), and flies expressing Lcp2-C99 under *Mhc* > *Gal4* appeared indistinguishable from wild type flies in wing posture, lifespan, etc., supporting the importance of APP signal sequence to ribosome stalling and toxicity in vivo.

We also tested whether co-translational membrane topogenesis of APP.C99 might contribute to its translational stalling. Charged residues flanking the TM domains are known to be involved in the “stop transfer” and membrane insertion of TM proteins [59]. Mutating three positively-charged K residues C-terminal of APP.C99 TM domain partially attenuated the internal stalling as indicated by the reduced lower band: upper band ratio (Additional file 1: Fig. S3g). Together, these data suggest that ER-targeting, translocon-gating, and subsequent protein folding and membrane insertion are events intrinsic to APP.C99 biogenesis and membrane topogenesis that cause ribosome stalling during its co-translational ER translocation.

### Engagement of co-translational quality control machinery to handle stalled translation of APP.C99

**Fig. 4:**
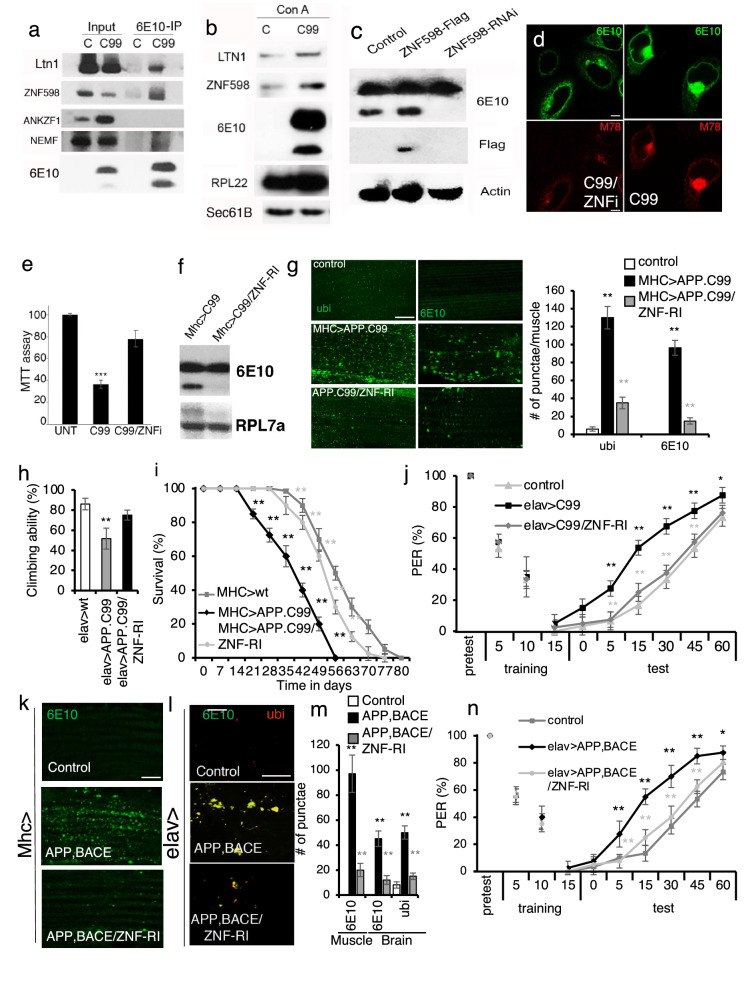
The ribosome stall sensor ZNF598 critically regulates the translational quality control and toxicity of APP.C99. **a** Immunoblots showing co-IP between APP.C99 and ZNF598 and other ribosome-associated QC factors. **b** Translocon pull-down with ConA beads showing increased ZNF598 and Ltn1 association with Sec61 translocon in APP.C99 transfected cells. **c** Immunoblots showing the effect of ZNF598 RNAi or OE on the level of stalled APP.C99. **d** Immunoblots showing the effect of ZNF598 RNAi on the level of mOC78-positive aberrant APP.C99 species. Scale bar, 5 µm. **e** MTT viability assay showing the effect of ZNF598 RNAi on APP.C99 toxicity. **f** Immunoblotd showing the effect of ZNF598 RNAi on the level of stalled APP.C99 in *Mhc* > *APP.C99* fly muscle. **g** Immunostaining and data quantification showing effect of ZNF598 RNAi on the level of Ub- and 6E10- positive aggregates in *Mhc* > *APP.C99* fly muscle (n = 10). Scale bar, 10 µm. **h** Effect of ZNF598 RNAi on locomotor activity in *elav* > *APP.C99* flies (n = 20–25). **i** Effect of ZNF598 RNAi on the lifespan of *Mhc* > *APP.C99* flies (n = 80–100). **j** Effect of ZNF598 RNAi on the aversive taste memory response in *elav* > *APP.C99* flies (n = 60). **k-m** Immunostaining (**k, l**) and data quantification (**m**) showing effects of ZNF598 RNAi on the level of 6E10-positive aggregates in the muscle of *Mhc* > *APP; BACE1* flies (**k,** n = 10), or effect of ZNF598 RNAi on the level of 6E10 and Ub double-positive aggregates in the brain of *elav* > *APP; BACE1* flies (**l,** n = 20–25). Scale bars: 10 µm (**k**), 20 µm (**l**). **n** Effect of ZNF598 RNAi on the aversive taste memory response in *elav* > *APP; BACE1* flies (n = 60–80). Error bars, ± SEM; ^∗^P < 0.05, ** P < 0.01. Immunoblots represent at least 3 independent repeats

To further dissect the molecular events associated with stalled translation of APP.C99, we performed immunoprecipitation (IP) of APP.C99 from HeLa cells and probed for the interaction with ribosome-mediated QC factors. Such interactions might occur due to either QC factor recruitment to stalled APP.C99 NPC/ribosome complex, or persistent association of QC factors with stalled NPCs after ribosomal release, as previously shown in yeast [33]. We detected robust APP.C99 interaction with ZNF598 (Fig. 4a), one of the earliest sensors of ribosome stalling and collision. Weak APP.C99 interaction with Ltn1 and NEMF, but no interaction with ANKZF1 (Fig. 4a), was also detected. We next analyzed ConA pull-down of Sec61 ER translocon for the presence of RQC factors, reasoning that, should the translation of APP.C99 be stalled on the ER during co-translational translocation, it would recruit the RQC machinery to the ER translocon. ZNF598, Ltn1, and ribosome subunit Rpl22 exhibited enhanced associated with Sec61B translocon in APP.C99-expressing cells (Fig. 4b). Thus, during co-translational import of APP.C99 to the ER, ribosome slowdown and collision occur, leading to recruitment of the RQC machinery in an attempt to resolve such stalling events.

### Rescue of APP.C99 toxicity by genetic manipulation of ribosome-mediated QC

We next tested the function of ribosome-mediated QC machinery in regulating APP.C99 expression and toxicity. As a sensor of ribosome stalling and collisions, ZNF598 ubiquitinates ribosomal small subunit proteins such as Rps10 of collided ribosomes to trigger disassembly of stalled ribosomes and QC of associated NPCs and mRNAs. In the absence of ZNF598 or its yeast counterpart Hel2, ribosomes would ignore stall signals, or that stalled ribosomes persist long enough and eventually complete translation [26, 61]. In APP.C99-transfected HeLa cells, ubiquitination of RPS10 was increased in a ZNF598-dependent manner (Additional file 1: Fig. S4a), indicating increased ribosome collision. Importantly, when ZNF598 was knocked down, the level of stalled APP.C99 was dramatically reduced (Fig. 4c). Conversely, ZNF598 overexpression moderately increased APP.C99 internal stalling in HeLa cells (Fig. 4c). APP.C99 stalled at the stop codon site was presumably also reduced by ZNF598 RNAi, and that the remaining APP.C99 signal likely corresponded to normal APP.C99 that had successfully finished translation and passed the translocon. Consistently, Pum-labeled stalled NPCs (Additional file 1: Fig. S4b), and mOC78-positive, aberrant APP.C99 species were greatly reduced by ZNF598 RNAi (Fig. 4d). The cytotoxicity of APP.C99 as measured by MTT assay was also reduced by ZNF598 RNAi (Fig. 4e).

We next used *Drosophila* models to test the effect of ribosome-mediated QC on APP.C99 translation and toxicity in vivo. RNAi of the fly ZNF598 homologue efficiently reduced APP.C99 internal stalling (Fig. 4f). A control RNAi transgene (*w* RNAi) had no such effect (Additional file 1: Fig. S4c). ZNF598 RNAi resulted in a reduction of APP.C99 aggregation as indicated by the decrease of Ub- and 6E10-positive signals (Fig. 4g), suggesting that it rescued proteostasis impairment caused by stalled APP.C99 products. Moreover, APP.C99-induced abnormal wing posture was effectively rescued by ZNF598 RNAi (Additional file 1: Fig. S4d). Boosting ribosome-mediated QC by OE of select co-translational QC factors, such as Pelo, Ltn1, Vms1/ANKZF1, and VCP also rescued APP.C99 induced wing posture defect (Additional file 1: Fig. S4d). At the whole organismal level, APP.C99-induced locomotor defect (Fig. 4h) and shortened lifespan (Fig. 4i) were both rescued by ZNF598 RNAi.

We further examined the effect of RQC manipulation on APP.C99 toxicity in neuronal settings. APP.C99-induced protein aggregation (Additional file 1: Fig. S4e) and neuronal degeneration (Additional file 1: Fig. S4f) were rescued by ZNF598 RNAi. The impairment of aversive taste memory caused by pan-neuronal expression of APP.C99 was also effectively rescued by ZNF598 RNAi (Fig. 4j).

We also tested the regulation by ZNF598 of APP.C99-related toxicity in the FL-APP context. For this purpose, we used *APP/BACE1* flies. Co-expression of APP/BACE1 in the muscle tissue led to decreased lifespan, which was rescued by ZNF598 RNAi (Additional file 1: Fig. S4g). This was correlated with reduced formation of 6E10-positive protein aggregates (Fig. 4k, m). Pan-neuronal expression of APP/BACE1 induced formation of 6E10- and ubiquitin-positive amyloid aggregates (Fig. 4l, m) and short-term memory defects as measured by the aversive taste memory behavior (Fig. 4n), which were both rescued by ZNF598 RNAi. Importantly, ZNF598 RNAi also rescued the early endosomal (Additional file 1: Fig. S4h) and lysosomal (Additional file 1: Fig. S4i) defects in APP.C99-expressing fly muscle tissues, supporting that stalled APP.C99 translation is the underlying cause of the endolysosomal and autophagic defects. Together, these data demonstrated that translation stalling is a major mechanism of APP.C99 toxicity at the cellular, tissue, and whole organismal levels, with the stalling sensor ZNF598 being a major regulator of such toxicity.

### Ribosome-mediated QC modulates disease phenotypes in the 5xFAD mouse model and RQC factors are found at the core of plaques from AD patient brains

**Fig. 5:**
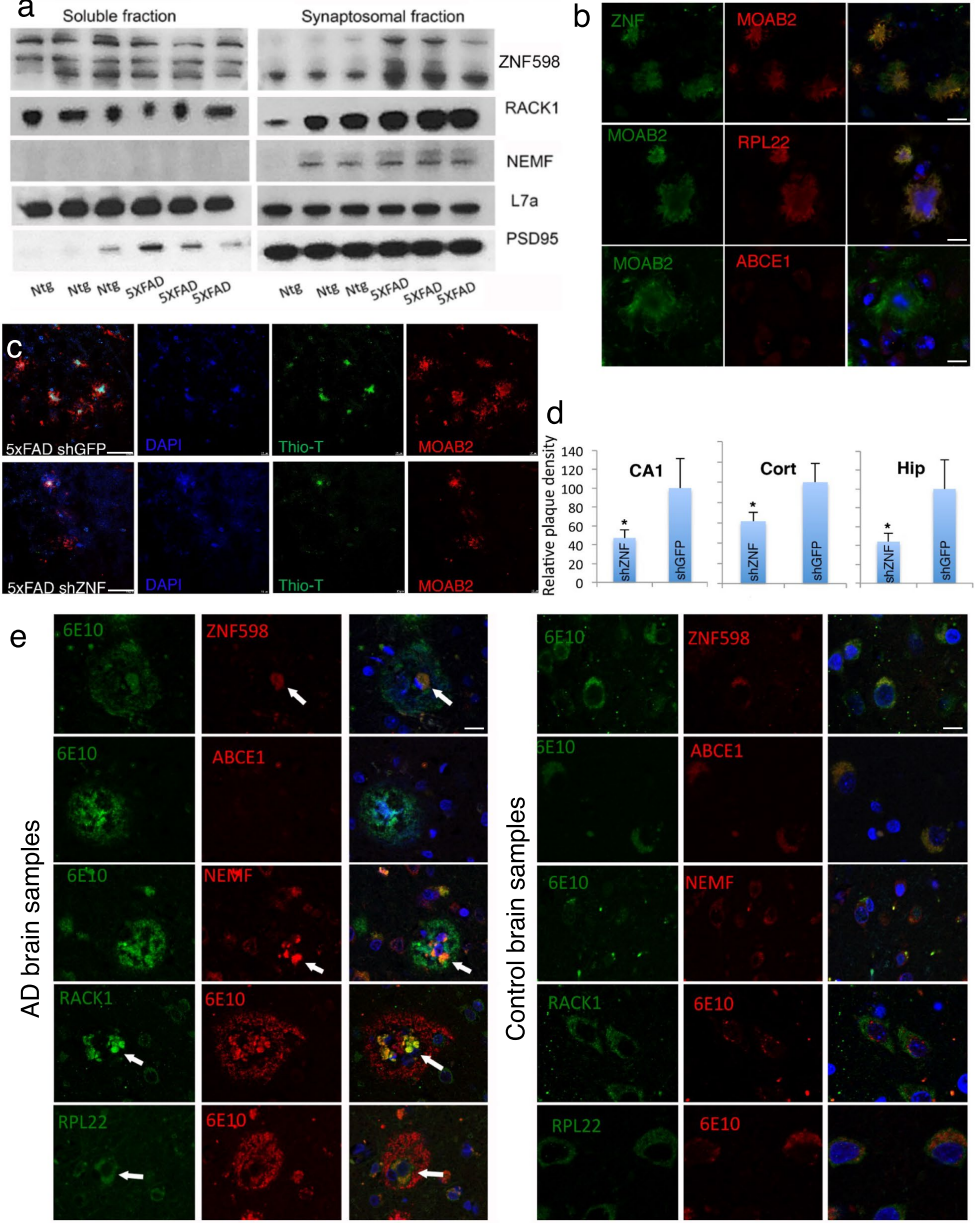
ZNF598 regulates disease phenotypes in 5xFAD mouse model. **a** Enrichment of ZNF598 in the synaptosome fraction of 5xFAD mouse brain. **b** Immunostaining showing colocalization of ZNF598 with ribosome large subunit protein Rpl22, but not ABCE1, in MOAB2-labelled plaques (n = 50). Scale bar, 5 µm. **c** Immunostaining showing the number and morphology of amyloid plaques in the cortex of 5xFAD mice treated with lenti-shGFP control (n = 10) or lenti-shZNF (n = 10). Scale bar, 10 µm. **d** Quantification of plaque density in the hippocampus (Hip), cortex (Cort), or CA1 region of the hippocampus (CA1). **e** Immunostaining of sections of AD brains (n = 3) showing deposition of ZNF598, NEMF, RPL22, and RACK1, but not ABCE1, to the core of amyloid plaques (n = 55–97). Scale bar, 5 µm. Error bars, ± SEM; ^∗^*P* < 0.05. Immunoblots represent at least 3 independent repeats

To obtain in vivo evidence for the relevance of translation stalling to AD pathogenesis in a mammalian system, we used the 5xFAD mouse model expressing human APP and PSEN1 with five FAD mutations: the Swedish (K670N/M671L), Florida (I716V), and London (V717I) mutations in APP and the M146L and L286V mutations in PSEN1. This commonly used AD model exhibits early and robust recapitulation of disease phenotypes associated with intracellular Aβ toxicity [62]. In 5xFAD mouse brain synaptosome fractions, we found increased levels of certain RQC factors, including ZNF598 and Rack1, another sensor of stalled ribosomes [24], suggesting the occurrence of ribosome collision/stalling and engagement of the RQC machinery (Fig. 5a). Intriguingly, ZNF598, but not ABCE1, was found in plaque-like structures in the hippocampal region of 3-month-old 5xFAD mice (Fig. 5b).

To examine the functional significance of ribosome stalling to AD pathogenesis in 5xFAD mice, we used lenti-viral injection into brain ventricles of newborn pups to knock down ZNF598, with the knockdown efficiency verified by WB (Additional file 1: Fig. S5a). Animals were analyzed at 3 months of age to look for earliest possible phenotypic outcomes. Compared to control injected animals, lenti-ZNF598 shRNA injected animals showed reduced number of amyloid plaque-like structures in the cortex and hippocampus (Fig. 5c, d). The plaques that did form were smaller, with less Thio-T-positive, β-sheet structure-containing materials in the core of the plaques (Fig. 5c, Additional file 1: S5b). Moreover, ZNF598 RNAi reduced Cathepsin D- and LAMP1-positive lysosomes (Additional file 1: Fig. S5c, d), suggesting that ZNF598 RNAi rescued lysosomal defects in 5xFAD mice. There was also a reduction of IBA1-positive microglia in the cortex of lenti-ZNF598 shRNA injected brain samples (Additional file 1: Fig. S5e). These data suggest that ribosome stalling plays an etiological role in the accumulation of defective APP translation products, which trigger lysosomal and autophagy defects, plaque formation, and neuroinflammation.

To further test the relevance of the RQC factors to AD pathogenesis, we stained control and human AD brains with antibodies against the RQC factors. Strikingly, certain RQC factors such as ZNF598, NEMF, and ANKZF1, were enriched in DAPI-positive structures at the core of amyloid plaques (Fig. 5e, Additional file 1: S5f), whereas others such as ABCE1 or eRF1, which were down-regulated by APP.C99 (Fig. 3a), were underrepresented in plaques (Fig. 5e, S5f). This result suggests that persistent ribosome stalling or failure to effectively resolve stalled ribosomes by the RQC machinery may contribute to plaque pathology by providing RQC remnants that seed amyloid plaque formation, supporting the importance of intracellular Aβ in AD pathogenesis [63–65] and the intracellular origin of amyloid plaques [55]. Interestingly, in mild cognitive impairment (MCI) patient brain samples, we observed nuclear localization of 6E10 positive and ZNF598 positive signals (Additional file 1: Fig. S5g), consistent with previous observation of stage-specific nuclear/perinuclear mOC78 positive amyloid signals [55] and suggesting disease stage-dependent modulation of APP.C99 and RQC factors.

### Potential role of CAT-tailed aberrant APP.C99 in seeding Aβ aggregation

**Fig. 6:**
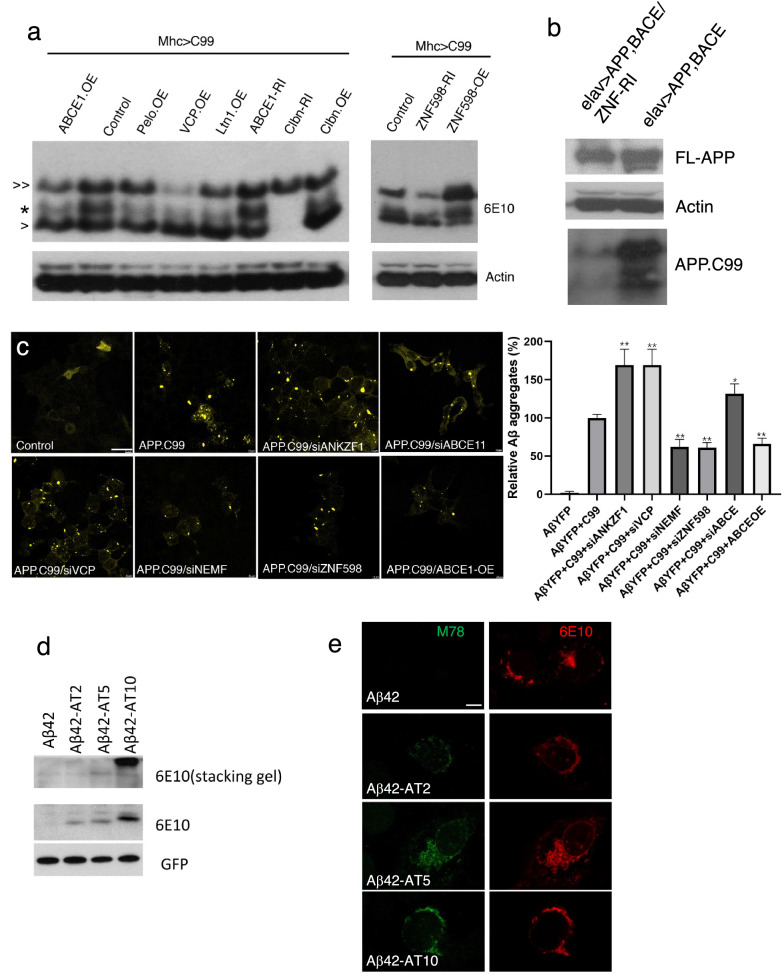
Ribosome stall-induced aberrant APP.C99 species seed amyloid β-42 aggregation. **a** Immunoblots showing effects of genetic manipulations of RQC and CAT-tailing related factors on the levels of the various APP.C99 species in fly muscle extracts. >  > : FL-APP.C99; * putative CAT-tailed, internally-stalled APP.C99; > : non-CAT-tailed, internally-stalled APP.C99. **b** Immunoblots showing the effect of ZNF598 RNAi on the level of stalled APP.C99 in *elav* > *APP; BACE1* fly brain extract. **c** Fluorescent images showing induction of Aβ42 aggregation by APP.C99 in HEK293 cells stably transfected with Aβ42-YFP, and the modifying effect of RNAi or OE of select RQC and CAT-tailing related factors. Fixed cells were directly imaged. Bar graph shows data quantification. Scale bar, 10 µm. **d** Immunoblots showing effect of artificial addition of different-lengthed AT tails on the aggregation of Aβ42. **e** Immunostainings showing effect of artificial addition of different-lengthed AT tails on the aggregation of Aβ42 in HeLa cells. 6E10 detects all Aβ42, whereas mOC78 detects aggregated Aβ42. Scale bar, 5 µm. Error bars, ± SEM; ^∗^*P* < 0.05, ^**^P < 0.01. Immunoblots represent at least 3 independent repeats

We further investigated the molecular mechanism underlying the seeding of amyloid plaque formation by defective APP.C99 RQC products. Subsequent to sensing of ribosome collision by ZNF598, stalled ribosomes are disassembled and NPCs still attached to 60S subunit are handled by the Ltn1/VCP/NEMF complex. In the process, stalled NPCs are modified by random addition of CAT-tails catalyzed by NEMF. To test whether translational stalling of APP.C99 resulted in CAT-tailing-like modification, we used high percentage Tris-Tricine gel with MES running buffer, which better separate CAT-tailed proteins [58]. With this method, we found that internally stalled APP.C99 expressed in fly tissues could be separated as a doublet, with the upper band running as a smear and close to the lower band (Fig. 6a), consistent with CAT-tailing-like modification [29, 35]. The level of the putative CAT-tailed species was increased by ZNF598 OE or ABCE1 RNAi but reduced by ZNF598 RNAi or ABCE1 OE (Fig. 6a). Moreover, RNAi of Clbn, the fly homolog of NEMF/RQC2 required for CAT-tailing [29, 58], dramatically reduced CAT-tailed and stalled APP.C99 (Fig. 6a), consistent with NEMF/Clbn being required for CAT-tailing and a stabilizing effect of CAT-tails on NPCs [35]. Overexpression of RQC factors VCP, Ltn1, and Clbn, or the no-go decay factor Pelo, which effectively rescued APP.C99-induced wing posture defect (Additional file 1: Fig. S4c), had variable effects on the expression levels of FL-APP.C99 and the unmodified stalled APP.C99, but invariably reduced the levels of the CAT-tailed form of stalled APP.C99 (Fig. 6a), supporting the notion that the CAT-tailed forms of stalled APP.C99 are the toxic species. In *elav* > *APP/BACE1* flies, APP.C99 produced from FL-APP also ran as upper and lower bands, and ZNF598 RNAi reduced the level of stalled and CAT-tailed APP.C99 (Fig. 6b), suggesting that regulation of APP.C99 translational stalling and CAT-tailing by ZNF598 also occurs in FL-APP context. In 5xFAD mouse brain samples, we also observed stalled and putative CAT-tailed APP.C99 (Additional file 1: Fig. S6a).

We next tested whether aberrant APP.C99 products resulting from the failed RQC process would seed Aβ aggregation. For this purpose, we used a HEK293 cell line stably expressing an Aβ42-YFP construct [37]. Under normal conditions, the Aβ42-GFP exhibits diffuse, low level cytoplasmic localization. However, when a seeding agent such as amyloid fibrils prepared from diseases brains was introduced, Aβ42-YFP formed prominent aggregates [37]. Introduction of ER-targeted APP.C99 (Additional file 1: Fig. S6b), but not APP.C99 without ER-targeting signal (Additional file 1: Fig. S6c), strongly promoted Aβ42-YFP aggregation. This APP.C99-induced Aβ42 aggregation was regulated by the RQC and CAT-tailing process, as RNAi of ZNF598 and NEMF, or ABCE1-OE greatly attenuated the ability of APP.C99 to induce Aβ42-YFP aggregation (Fig. 6c), whereas RNAi of ANKZF1 and VCP, factors required for the release of stalled NPCs from 60S ribosome [66] or turnover of CAT-tailed NPCs [33], respectively, or the RNAi of ABCE1, facilitated the ability of APP.C99 to induce Aβ42-YFP aggregation (Fig. 6c). These data support the notion that aberrant RQC products, likely the CAT-tailed APP.C99 species, help seed Aβ42-YFP aggregation. To further test this hypothesis, we appended artificial CAT-tails to Aβ42 to mimic CAT-tailing of internally stalled APP.C99 and observed tail length-dependent facilitation of Aβ42 aggregation as detected by WB analysis as SDS-resistant high MW species trapped in the stacking gel (Fig. 6d), and by immunostaining with the aggregate-specific mOC78 antibody (Fig. 6e) or Amytracker (Additional file 1 1: Fig. S6d). This was correlated with tail length-dependent Aβ42 toxicity (Additional file 1: Fig. S6e). We also appended artificial CAT-tail to “normal” FL-APP.C99 and observed enhanced aggregation (Additional file 1: Fig. S6f). Together, these data demonstrate CAT-tail-mediated facilitation of aggregation and toxicity by aberrant APP.C99 species.

## Discussion

Development of effective AD-modifying therapeutics requires the identification of the earliest pathobiology and the causative factors. This has been a persistent and elusive goal in the field. Previous studies have heavily focused on Aβ, initially on extracellular Aβ and later intracellular Aβ. This study highlights an unanticipated etiological role of CAT-tailed APP.C99 species resulting from ribosome collision/stalling during the synthesis of APP, a step earlier than the processing of post-synthesis, mature APP. Although RQC is a newly discovered process studied primarily in yeast, its importance to human health is beginning to be appreciated. Mutations in genes involved in RQC, such as Ltn1, HBS1, and NEMF, cause neurodegeneration phenotypes in the mouse [67–69]. Recent work revealed that RQC is important for quality control of mitochondrial outer membrane-associated ribosomes [58, 70], and that inefficiency of this process can compromise mitochondrial homeostasis and contribute to the pathogenesis of Parkinson’s disease [58] and C9orf72-associated ALS/FTD [35]. These studies revealed that mitochondrial stress can impair the RQC process by destabilizing the translation termination and ribosome recycling factors including ABCE1, which is rate-limiting in cells [71], resulting in CAT-tailing-like protein C-term extension known as MISTERMINATE [58]. Such feedback regulation offers a mechanistic link between proteostasis failure and mitochondrial dysfunction, two pathological hallmarks common to all neurodegenerative disease [72]. As APP.C99 selectively accumulates in vulnerable neurons in AD [73] and is known to show increased presence at mitochondria-associated ER membranes [74], and β-cleavage activity is present at the ER [75–77] or the endosomes [78], our finding of RQC manipulation being effective in rescuing APP.C99-related toxicity in both APP.C99 and FL-APP contexts supports that inefficient QC of ER-associated stalled translation of APP is critically involved in the generation of toxic APP.C99 species during AD pathogenesis. We note that the ER is key in the generation of aberrant APP/APP.C99 but it does not necessarily have to be the only site of processing or action of CAT-tailed APP or APP.C99 in the disease process. The lysosomes and endosomes or other organelles may also be involved. ER stress is an early event in AD and other diseases, we propose that inefficient ER-associated RQC may provide a mechanistic link between proteostasis failure and ER dysfunction. It remains to be determined whether MISTERMINATE-related mechanism may contribute to the ABCE1 and eRF1/3 deficits in APP.C99-expressing cells and whether altered ER-mitochondrial communication is involved. In this respect, it is worth mentioning that APP.C99 has been shown to elicit mitochondrial structure, function, and mitophagy defects [79].

**Fig. 7:**
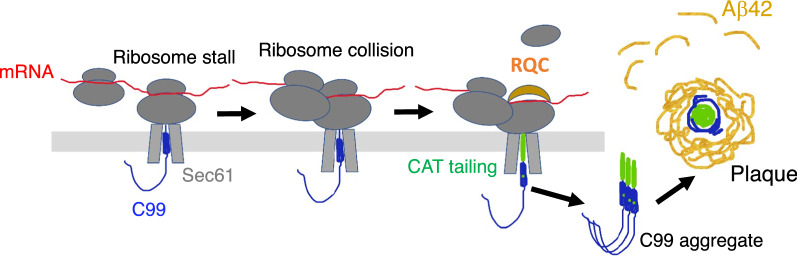
Working Model. Diagram depicting events from ribosome stalling at ER translocon to the generation of CAT-tailed aberrant APP.C99 species that seed amyloid plaque formation

Despite its initial microscopic discovery more than a century ago by Dr. Alois Alzheimer, the origin of amyloid plaques and key factor(s) that seed plaque formation are still being hotly debated [80], with extracellular Aβ, intracellular Aβ, and oligomeric Aβ all being implicated. The fact that AD brain extracts or extracts from cells pre-seeded with AD brain extracts are more potent than in vitro formed Aβ oligomers in seeding amyloid plaque formation in vivo supports the involvement of other cellular factors in seeding amyloid plaques [81]. Our results, especially the observation of DAPI-positive and RQC factor-positive materials in the center of amyloid plaques found in AD patient brain samples, and the ability of aberrant, CAT-tailed APP.C99 to seed Aβ-42 aggregation, support the idea proposed more than a century ago that amyloid plaques originate from within degenerating neurons or neuronal processes [80]. We hypothesize that intrinsic features of APP.C99 translation, including ER targeting, co-translational translocation, co-translational folding, and membrane topogenesis, slow down translation and impose higher demand for co-translational quality control activities. Inadequacy of ribosome-mediated QC machinery, or exacerbated ribosome collision by environmental stress [82], can result in persistent ribosome collision/stalling and accumulation of faulty APP.C99 translation products with CAT-tails. If not cleared in a timely manner, these aberrant APP.C99 species will lead to degeneration of neuronal cell body or neurites. Due to the unusual stability or aggregation properties conferred by the CAT-tails, these aberrant APP.C99 species will persist after the demise of the host cell, recruiting metastable proteins such as Aβ from the host cell and surrounding cells to seed extracellular plaque formation (Fig. 7). Although our use of artificial AT repeats is sufficient to mimic the effect of aberrant APP.C99 in facilitating Aβ-42 aggregation, future biochemical studies will determine the nature of the CAT-tails formed on APP.C99 in AD condition, as amino acids other than A and T can be incorporated into CAT-tails in flies and mammalian cells [35, 58].

Abnormalities in the endolysosomal and autophagy systems are prominent features of AD, predating the appearance of plaque and NFT pathologies [53]. Genome-wide association studies have linked variants in genes in the endolysosomal and autophagy systems to risks of developing AD [13]. Independent studies using different experimental systems have implicated APP.C99 instead of Aβ as causative of the endolysosomal and autophagy defects in AD. However, it remains controversial whether the endolysosomal and autophagy defects are cause or consequence of the primary drivers of the disease process. In our models, we found early endosomal defects, as well as lysosomal and autophagy defects in the fly AD models. More importantly, genetic manipulations that reduced the translational stalling of APP.C99 rescued disease phenotypes concomitant with rescue of the endolysosomal and autophagy defects, supporting the notion that the endolysosomal and autophagy defects are caused by the ribosome stalling process, or the resulting CAT-tailed APP.C99. We showed that restoring the endolysosomal and autophagic system function is sufficient to rescue APP.C99 induced disease phenotypes, suggesting that endolysosomal and autophagy defects are key mediators of APP.C99 pathogenesis. Future studies will also address how the aberrant RQC products of APP.C99 impair endolysosomal and autophagy systems. As these systems are involved in clearing aberrant APP.C99, their gradual impairment during disease or aging will eventually result in a pathological buildup of toxic APP.C99 species.

Given the emerging evidence supporting APP.C99 as an etiological trigger of AD pathology, interventions targeting APP.C99 or its downstream effects on the endolysosomal and autophagy systems may be considered for therapeutic purposes. With data casting doubts on the therapeutic prospect of targeting APP-processing enzymes, therapeutic approaches that restore APP.C99 homeostasis by other means are warranted. In this regard, modulating ribosome-mediated QC to get rid of aberrant APP.C99 RQC products, or restoring endolysosomal and autophagy function impaired by aberrant APP.C99 deserve serious consideration, as such manipulations rescued AD-related synapse and network dysfunction and behavioral outcomes in our AD model studies. Future studies in patient iPSC-derived AD models will test whether our findings in transgenic animal models also apply to disease models with more physiological APP expression. Finally, boosting ER and mitochondrial function, which are expected to indirectly reduce the ribosome stalling of ER translocon-engaged APP.C99, may also prove to be beneficial.

In conclusion, despite the fact that genetic evidence strongly supports aberrant APP metabolism in APP pathogenesis, the pathogenic protein species derived from APP and their sites of action remain incompletely defined. Our results identify APP as a physiological substrate of the RQC process whose intrinsic properties of translational regulation and membrane topogenesis entail higher RQC activities. Our results further show that amyloid plaque formation is the consequence and manifestation of a deeper level proteostasis failure caused by translationally stalled and CAT-tailed APP.C99 species, previously unrecognized etiological drivers of AD that are prone to aggregation and cause endolysosomal and autophagy defects and possibly other cytopathologies of AD. We anticipate   that discovery of drugs that target the translational stalling, RQC and CAT-tailing processes, or those that compensate for the endolysosomal and autophagic defects caused by aberrant APP.C99 may  lead to novel therapeutics for AD and related diseases.

## Supplementary Information

**Additional file 1:** Supplementary figures and supplementary table 1.

## Data Availability

The datasets used and/or analyzed during the current study are available from the corresponding author on reasonable request.
