# Supplementary material for: Inefficient quality control of ribosome stalling during APP synthesis generates CAT-tailed species that precipitate hallmarks of Alzheimer’s disease
Source: Acta Neuropathol Commun. 2021 Oct 18;9:169. doi: 10.1186/s40478-021-01268-6 (PMC8522249; doi:10.1186/s40478-021-01268-6)
Supplement: Supplementary file 1 — Additional file 1: Supplementary figures and supplementary table 1. [file 40478_2021_1268_MOESM1_ESM.pdf]

Supplementary information

Fig. S1 Endolysosomal and autophagy defects contribute to APP.C99 toxicity in *Drosophila* neuromuscular tissues. **a** Effect of APP.C99 on mito-Ca^{2+} level in adult fly muscle (n=10) as monitored with mito-GCaMP. Bar graph shows data quantification. Scale bar, 5 µm. **b** Effect of neuronal expression of APP.C99 on synaptic marker expression at the larval NMJ. The synapse of segment A3, muscle 6/7 was stained with presynaptic neuronal marker HRP and postsynaptic marker Dlg. Merged images is on the right. Dlg expression was greatly reduced. Scale bar, 10 µm. **c** Aversive taste memory assay in control flies and transgenic flies co-expressing FL-APP and BACE1 pan-neuronally (n=60-80). **d** Immunostaining showing effect of neuronal APP.C99 expression on proteostasis as detected with anti-Ub staining of fly brain tissue (n=10). Bar graph shows data quantification. Scale bar, 20 µm. **e** Immunostaining showing effects of pan-neuronal APP.C99 expression on TH^{+} DA neuron number in the PPL cluster of adult fly brains (n=10). Bar graph shows data quantification. Scale bar, 20 µm. **f** Effect of APP.C99 expression on mito-Ca^{2+} level as monitored with mito-GCaMP in brain samples (n=10). To specifically detect mito-Ca^{2+} in DA neurons, the *TH-Gal4* driver was used. Bar graph shows data quantification. Scale bar, 20 µm. **g** Accumulation of enlarged early endosomes as detected with anti-Rab5 antibody in fly brain expressing APP.C99 (n=10). Bar graph shows data quantification. Scale bar, 20 µm. **h** Immunostaining showing localization of APP.C99 in LAMP1-positive lysosomes in fly muscles (n=10) expressing APP.C99. Scale bars, 5 µm. **i, j** Effect of APP.C99 expression on perinuclear accumulation of LC3 (**i**) and P62 (**j**) in HeLa cells. Scale bar, 10 µm. **k**, Western blot analysis showing the effect of APP.C99 overexpression on the expression of LC3 and p62 proteins in HeLa

cells with and without bafilomycin (BafA1) treatment. **l** Effect of co-expression of Hsp70, Mitf, Fip200, ATG1, and Ref2p/p62 on FL-APP-induced wing posture defect (n>80). Data are representative of at least 3 independent experiments. Error bars, ± SEM; * P< 0.05, ** P<0.01 in Student's *t*-tests. ns, not significant.

Fig. S2 Ribosome stalling during co-translational ER translocation of APP.C99. **a, b** MTT cell viability assay of HeLa cells transfected with the indicated plasmids. **c** Immunostaining showing co-localization of APP.C99 with the early endosome marker Rab5 and lysosome marker CTSD in perinuclear structures marked with arrows in HeLa cell. Scale bar, 10 µm. **d** Immunoblots showing the presence of both APP.C99 species in ConA beads-captured ER translocon complex, and their release into the supernatant after EDTA or RNase A treatment. Untreated samples on the right showed residual amount of release in buffer alone during the treatment conditions. **e** Diagram of APP.C99, with the locations of the 6E10 antigen, Aβ42 region, the TM domain, the 3K to 3A mutations, and the HA insertions indicated. **f** Western blot analysis showing the effect of the location of HA insertion site on the ability of the internally stalled protein to contain HA epitope (marked with an arrow). Note that the weak HA signal in ER-C99-SK-HA was due to degradation of the full-length protein and was also detected by the 6E10 antibody. Error bars, ± SEM; ** P<0.01, *** P < 0.001. Immunoblots represent at least 3 independent repeats.

Fig. S3. ER targeting and ABCE1 deficiency contribute to stalled translation of APP.C99. **a** Immunostaining showing the effect of ABCE1 OE or RNAi on Pum-labelled stalled NPCs in APP.C99 transfected HeLa cells. Scale bar, 10 µm. **b** Immunostaining showing the effect of ABCE1 OE on

the level of perinuclear APP.C99 detected with 6E10 in HeLa cells. Scale bar, 10 µm. **c**, Western blot analysis showing effect of ABCE1 RNAi on the expression of LC3 and p62 proteins in HeLa cells overexpressing APP.C99. **d** Immunostaining of fly larval NMJ showing effect of neuronal ABCE1 OE on the level of synaptic marker expression in *elav>APP;BACE1* fly larvae (n=10). The synapse of segment A3, muscle 6/7 was stained with presynaptic neuronal marker Hrp and postsynaptic marker Dlg. Merge image is on the right. Reduced Dlg level caused by APP and BACE1 co-expression was rescued by ABCE1 OE. Bar graph shows data quantification. Scale bar, 10 µm. **e** Effect of ABCE1 OE on the memory defect in *elav>APP; BACE1* flies (n=60-80). **f** Immunostainings showing the effect of removing the signal sequence on the level of mOC78 positive APP.C99 signal in HeLa cells transfected with APP.C99 with or without the signal sequence. Scale bar, 10 µm. **g** Immunoblots and quantification showing the effect of K to A mutations on APP.C99 translational stalling. Error bars, ± SEM; ** P<0.01. Immunoblots represent 2 repeats.

Fig. S4 The ribosome stalling sensor ZNF598 critically regulates the translational quality control and toxicity of APP.C99. **a** Immunoblots showing increased ubiquitination of Rps10 in APP.C99 transfected cells and the inhibitory effect of ZNF598 RNAi on such ubiquitination. **b** Immunostaining showing effect of ZNF598 RNAi on Pum-labelled stalled NPCs in APP.C99 transfected cells. Scale bar, 5 µm. **c** Immunoblots showing lack of effect of a *w* RNAi transgene on translationally stalled and total APP.C99 levels. **d** Effect on APP.C99-indiced wing posture defects by the indicated genetic modifiers (n=80). **e** Immunostaining showing effect of ZNF598 RNAi on the level of 6E10 and Ub double-positive aggregates in *elav>APP.C99* transgenic fly

brains. Bar graph shows data quantification. Scale bar, 20 µm. **f** Immunostaining showing effect of ZNF598 RNAi on the number of TH^{+} DA neurons in the PPL cluster of *elav>APP.C99* fly brain. Bar graph shows data quantification (n=10-15). Scale bar, 20 µm. **g** Effect of ZNF598 RNAi on the lifespan of *Mhc>APP; BACE1* flies (n=80). **h** Effect of ZNF598 RNAi on Rab5-positive early endosomes in *Mhc>APP.C99* flies (n=10). Bar graph shows data quantification. Scale bar, 5 µm. **i** Effect of ZNF598 RNAi on LAMP1-positive lysosomes in *Mhc>APP.C99* flies (n=10). Bar graph shows data quantification. Scale bar, 20 µm. Error bars, ± SEM; ** P<0.01. Immunoblots represent at least 3 independent repeats.

Fig. S5 ZNF598 regulates disease phenotypes in the 5xFAD mouse model. **a** Immunoblots showing effect of Lenti-shZNF injection on ZNF598 protein level in whole brain extracts. shZNF#2, which showed effect knockdown effect, was chosen for further experiment. **b** Images showing effect of Lenti-shZNF on the number of Thio-T positive amyloid plaques in different brain regions. Scale bar, 10 µm. **c-e** Data quantification showing effect of ZNF598 RNAi on CTSD-positive (**c**), and LAMP1-positive lysosomes (**d**), or IBA1-positive microglia (**e**) in brain sections (n=5). Scale bar, 10 µm. **f** Quantification of the number of amyloid plaques (n=75-97) found in human AD brain samples that are positive or negative for the indicated RQC factors. Error bars, ± SEM; ** P<0.01, *** P < 0.001. **g**, Immunofluorescence images showing the localization of 6E10-positive amyloid signals and ZNF598 signals in MCI patient brain samples. Arrows mark nuclear localization. Immunoblots represent at least 3 independent repeats.

Fig. S6 Ribosome stall-induced aberrant APP.C99 seeds amyloid β-42 aggregation. **a**, Immunoblots showing the detection of C99 with 6E10 antibody or C99 and C83 with the APP C-term antibody (C1/6.1) in synaptosomal fractions of control or 5xFAD mouse brain samples. Synaptic protein PSD95 serves as loading control. The diffuse C99-stall band likely contains CAT-tailed and non-CAT-tailed species. **b** Immunostainings showing colocalization of ER-Flag-C99 with Aβ42-YFP in HEK293 cells stably transfected with Aβ42-YFP. Arrow marks a large Aβ42-YFP aggregate. Scale bar, 10 μm. **c** Immunostaining showing lack of effect on Aβ42-YFP aggregation by APP.C99 without signal sequence (No ER-Flag-C99). Scale bar, 10 μm. **d** Fluorescent images showing effect of artificial addition of different-lengthed AT tails on the aggregation of Aβ42 in HeLa cells as detected with the aggregation-specific dye Amytracker. Scale bar, 5 μm. **e** MTT cell viability assays (n=4) showing effect of artificial addition of different-lengthed AT tails on the toxicity of Aβ42 in HeLa cells. **f**, Immunofluorescence images showing the effect of addition of artificial CAT-tail (AT5) on the aggregation of APP.C99 as detected with Amytracker or mOC78 immunostaining. Error bars, ± SEM; ∗P< 0.05, ** P<0.01, *** P< 0.001.

Table S1 Individual demographic, pathological, and clinical details of the AD and control cohorts used.

Figure S1

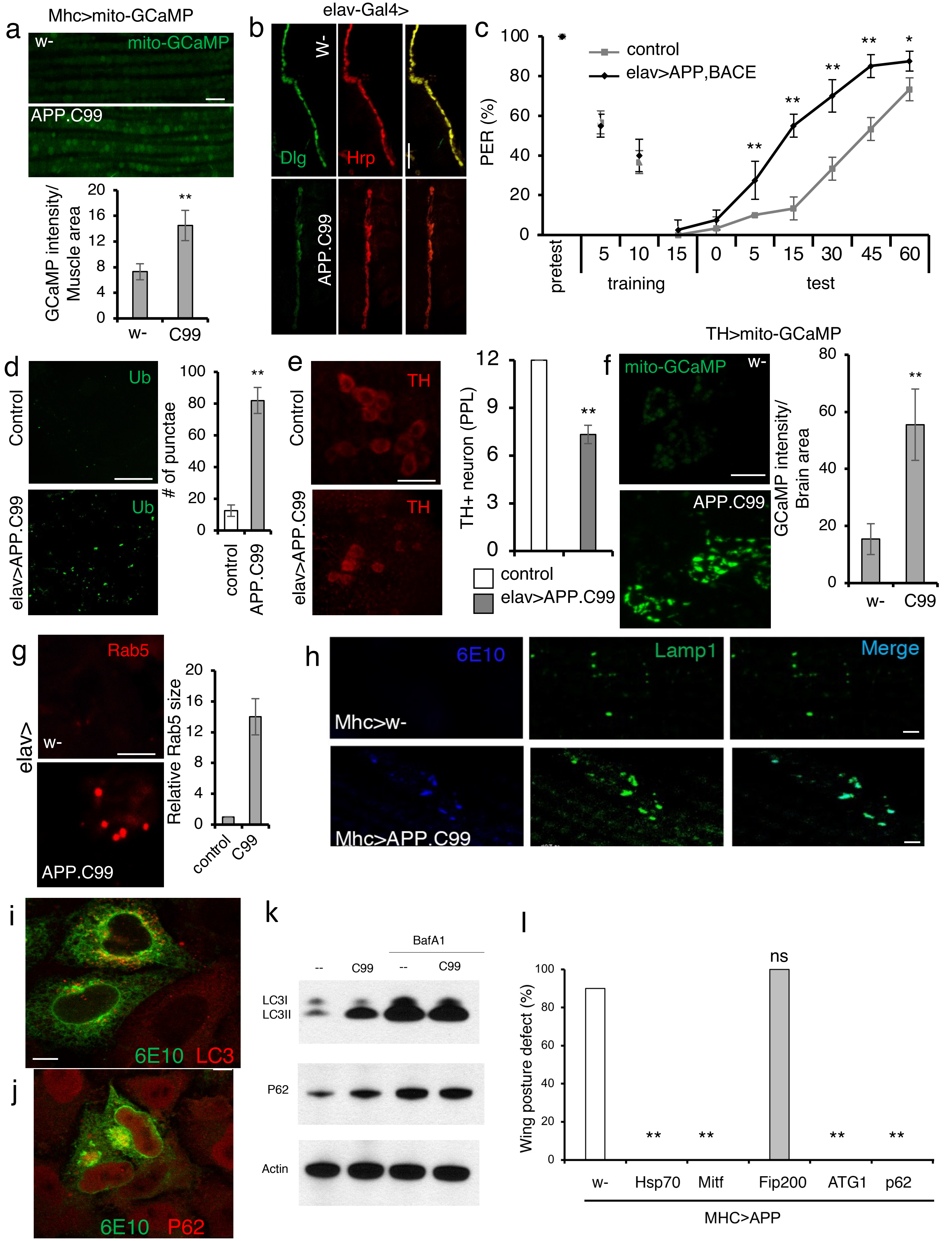

a
Mhc>mito-GCaMP
w-
mito-GCaMP
APP.C99
GCaMP intensity/ Muscle area
w-
C99
b
elav-Gal4>
W-
Dlg
Hrp
APP.C99
c
control
elav>APP,BACE
PER (%)
pretest
training
test
d
Control
Ub
elav>APP.C99
# of punctae
control
APP.C99
e
Control
TH
elav>APP.C99
TH+ neuron (PPL)
control
elav>APP.C99
f
TH>mito-GCaMP
mito-GCaMP
w-
APP.C99
GCaMP intensity/ Brain area
w-
C99
g
Rab5
elav>
w-
APP.C99
Relative Rab5 size
control
C99
h
6E10
Lamp1
Merge
Mhc>w-
Mhc>APP.C99
i
6E10 LC3
j
6E10 P62
k
BafA1
--
C99
LC3I
LC3II
P62
Actin
l
Wing posture defect (%)
ns
w-
Hsp70
Mitf
Fip200
ATG1
p62
MHC>APP

Figure S2

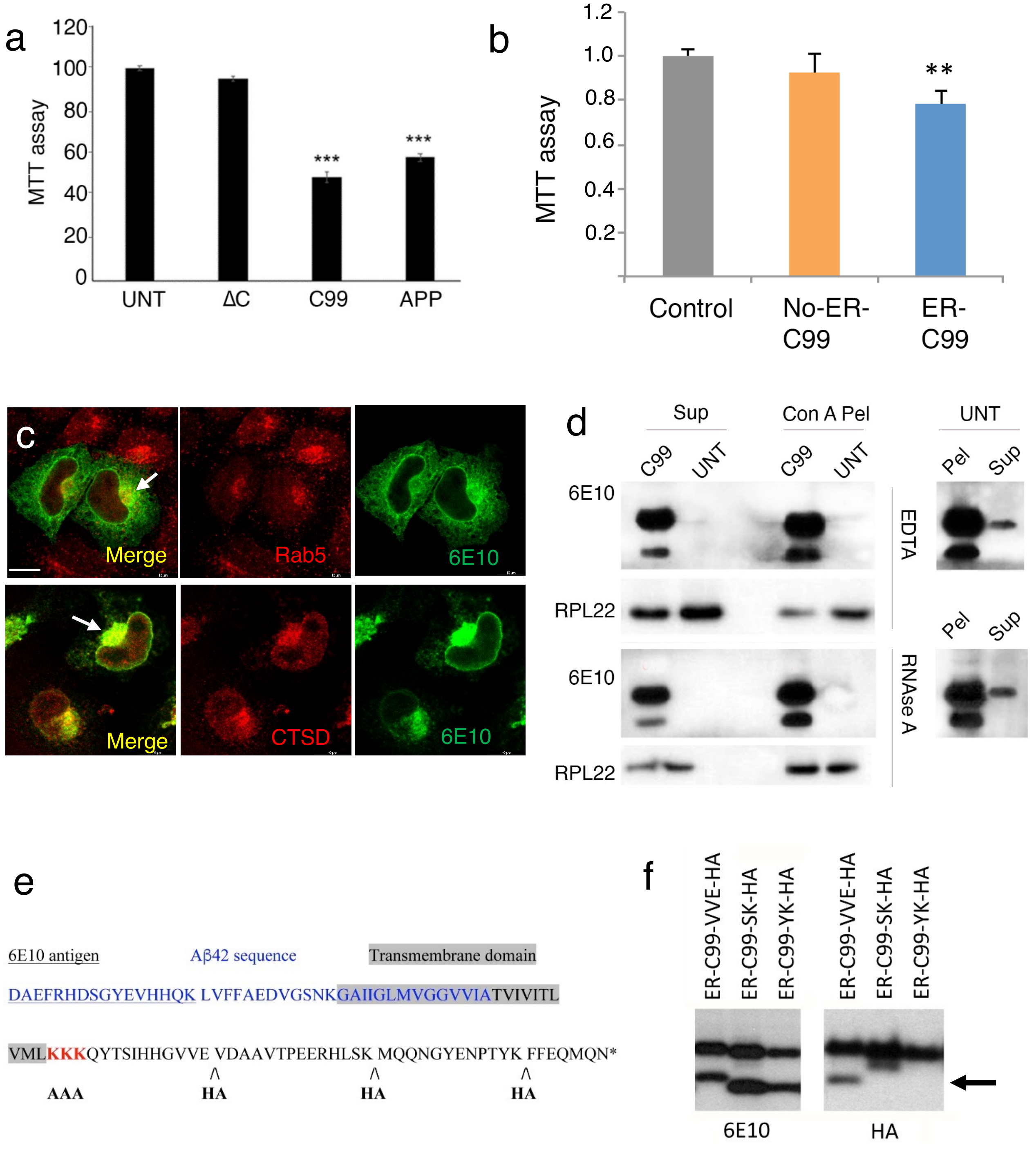
a
MTT assay
0
20
40
60
80
100
120
UNT
ΔC
C99
APP
***
***
b
MTT assay
0.2
0.4
0.6
0.8
1.0
1.2
Control
No-ER-C99
ER-C99
**
c
Merge
Rab5
6E10
Merge
CTSD
6E10
d
Sup
Con A Pel
UNT
C99
UNT
C99
UNT
Pel
Sup
6E10
RPL22
EDTA
6E10
RPL22
RNAse A
Pel
Sup
e
6E10 antigen
Aβ42 sequence
Transmembrane domain
DAEFRHDSGYEVHHQK LVFFAEDVGSNKGAIIGLMVGGVVIATVIVITL
VMLKKKQYTSIHHGVVE VDAAVTPEERHLSK MQQNGYENPTYK FFEQMQN*
AAA
HA
HA
HA
f
ER-C99-VVE-HA
ER-C99-SK-HA
ER-C99-YK-HA
ER-C99-VVE-HA
ER-C99-SK-HA
ER-C99-YK-HA
6E10
HA

Figure S3

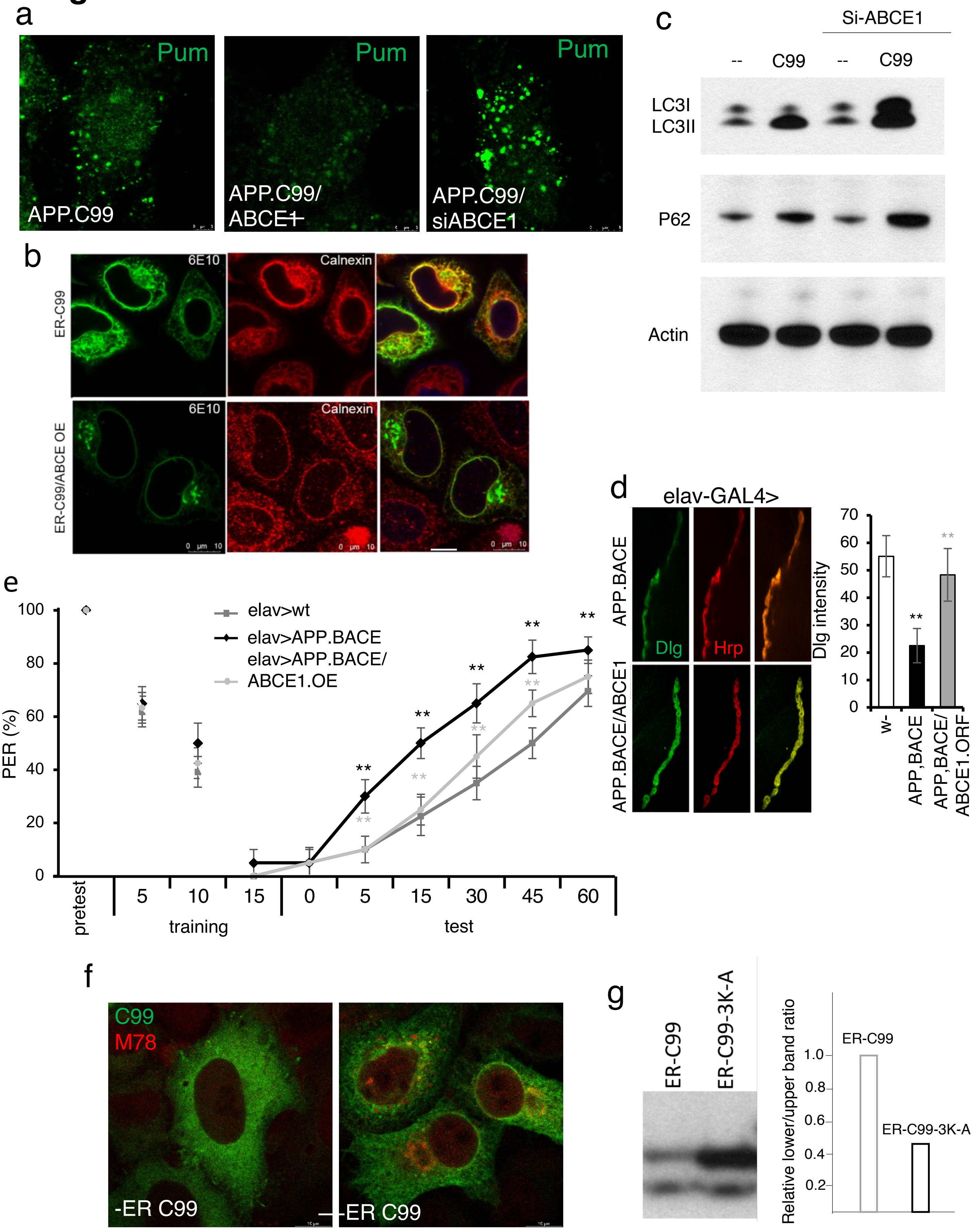
a
Pum
APP.C99
APP.C99/
ABCE1
APP.C99/
siABCE1
b
ER-C99
ER-C99/ABCE OE
6E10
Calnexin
c
Si-ABCE1
--
C99
LC3I
LC3II
P62
Actin
d
elav-GAL4>
APP.BACE
APP.BACE/ABCE1
Dlg
Hrp
Dlg intensity
w-
APP,BACE
APP,BACE/
ABCE1.ORF
e
elav>wt
elav>APP.BACE
elav>APP.BACE/
ABCE1.OE
PER (%)
pretest
training
test
f
C99
M78
-ER C99
ER C99
g
ER-C99
ER-C99-3K-A
Relative lower/upper band ratio

Figure S4

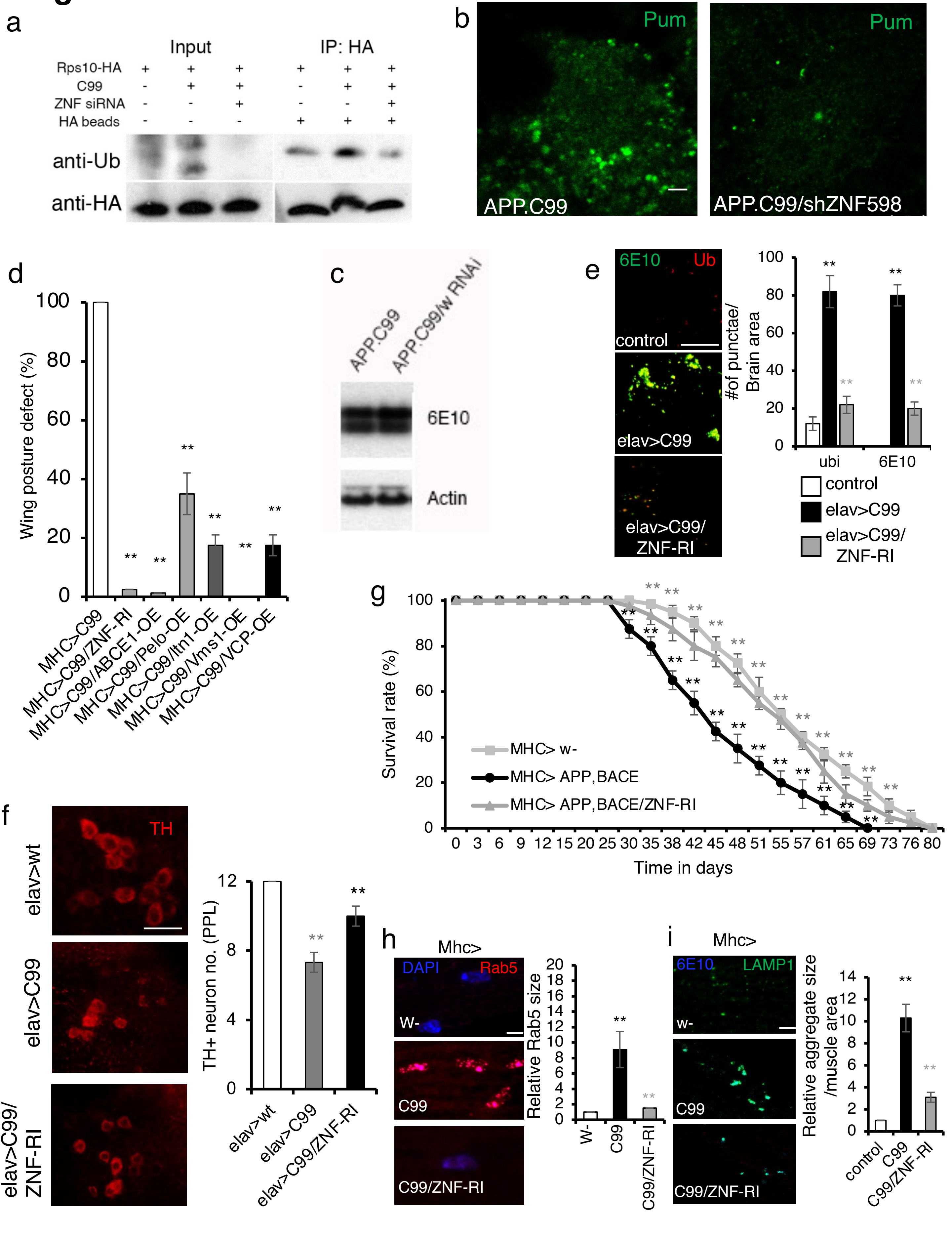

a
Input
IP: HA
Rps10-HA
C99
ZNF siRNA
HA beads
anti-Ub
anti-HA
b
Pum
APP.C99
APP.C99/shZNF598
c
APP.C99
APP.C99/w RNAi
6E10
Actin
d
Wing posture defect (%)
MHC>C99
MHC>C99/ZNF-RI
MHC>C99/ABCE1-OE
MHC>C99/Pelo-OE
MHC>C99/ltn1-OE
MHC>C99/Vms1-OE
MHC>C99/VCP-OE
e
6E10
Ub
control
elav>C99
elav>C99/ZNF-RI
#of punctae/Brain area
ubi
6E10
g
Survival rate (%)
Time in days
MHC> w-
MHC> APP,BACE
MHC> APP,BACE/ZNF-RI
f
TH
elav>wt
elav>C99
elav>C99/ZNF-RI
TH+ neuron no. (PPL)
h
Mhc>
DAPI
Rab5
W-
C99
C99/ZNF-RI
Relative Rab5 size
i
Mhc>
6E10
LAMP1
w-
C99
C99/ZNF-RI
Relative aggregate size /muscle area
control

Figure S5

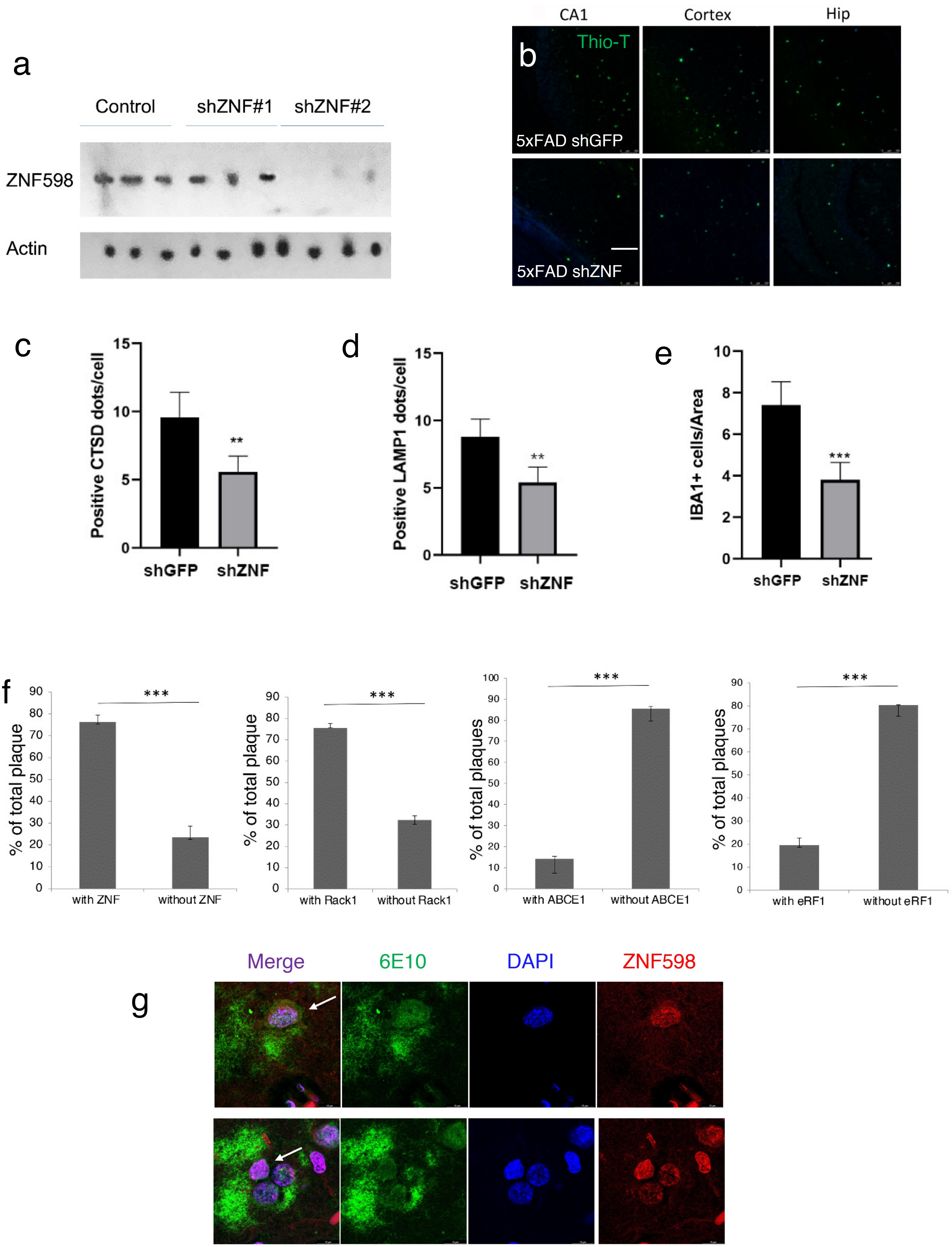
a
Control
shZNF#1
shZNF#2
ZNF598
Actin
b
CA1
Cortex
Hip
Thio-T
5xFAD shGFP
5xFAD shZNF
c
Positive CTSD dots/cell
shGFP
shZNF
d
Positive LAMP1 dots/cell
shGFP
shZNF
e
IBA1+ cells/Area
shGFP
shZNF
f
% of total plaque
with ZNF
without ZNF
with Rack1
without Rack1
% of total plaques
with ABCE1
without ABCE1
with eRF1
without eRF1
g
Merge
6E10
DAPI
ZNF598

Figure S6

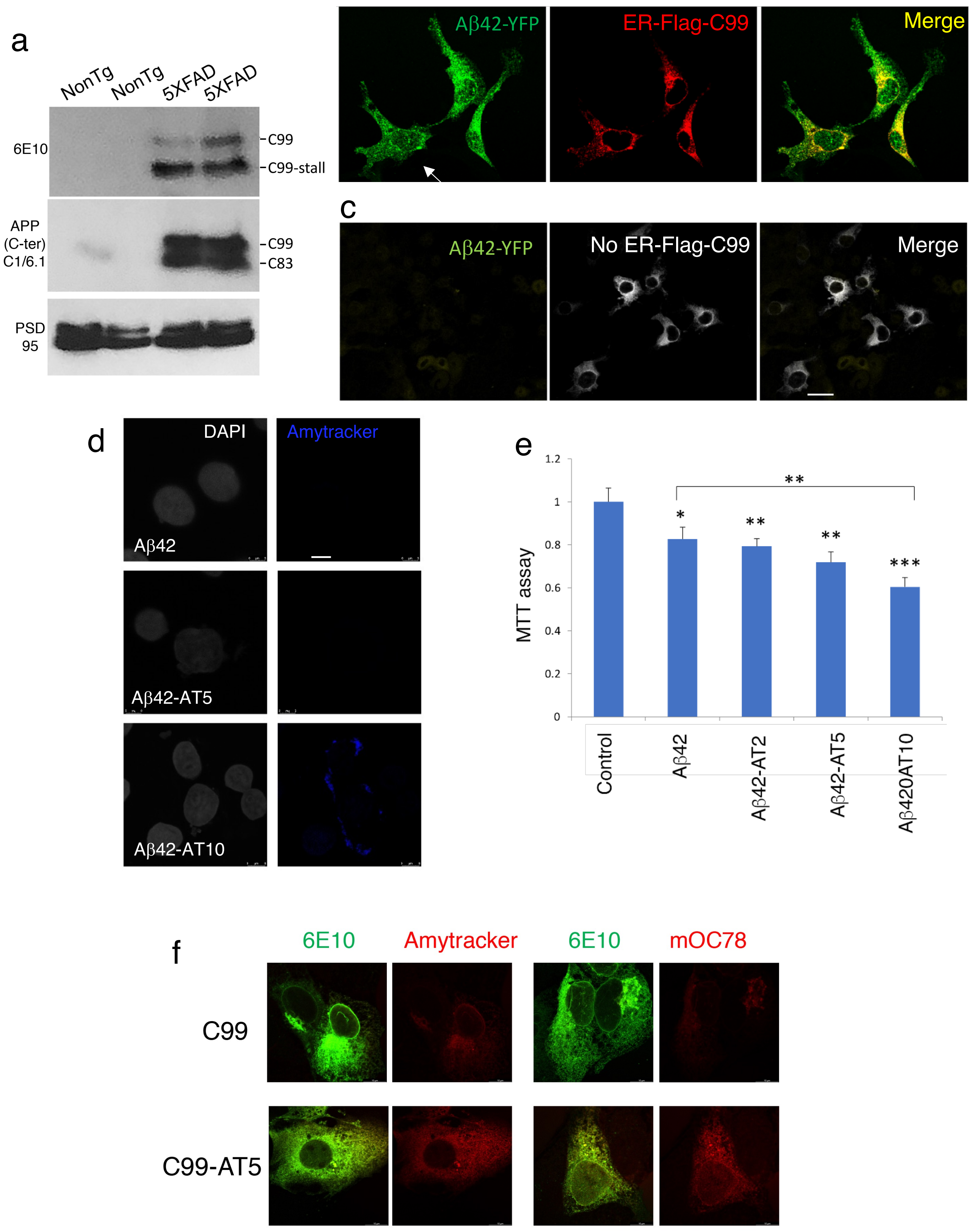
a
NonTg
NonTg
5XFAD
5XFAD
6E10
C99
C99-stall
APP (C-ter) C1/6.1
C99
C83
PSD 95
b
Aβ42-YFP
ER-Flag-C99
Merge
c
Aβ42-YFP
No ER-Flag-C99
Merge
d
DAPI
Amytracker
Aβ42
Aβ42-AT5
Aβ42-AT10
e
MTT assay
1.2
1
0.8
0.6
0.4
0.2
0
Control
Aβ42
Aβ42-AT2
Aβ42-AT5
Aβ420AT10
*
**
**
***
**
f
6E10
Amytracker
6E10
mOC78
C99
C99-AT5

Supplementary Table S1

Case P#	ADNC level	Sex	Age at death	Clinical	Primary Neuropath Dx	PMI (hrs)	Frozen Block Label	Frozen Block Side	Frozen Block Region
P2850	Not	F	92	Control	AGD, limbic	8.0	Z5	L	MFG
P2850	Not	F	92	Control	AGD, limbic	8.0	Z6	L	ITG
P2947	Low	M	95	Control	None	9.6	Z2	L	MFG
P2947	Low	M	95	Control	None	9.6	Z3	L	ITG
P3006	Low	M	91	Control	None	5.6	X4	L	MFG
P3006	Low	M	91	Control	None	5.6	Z2	L	ITG
P2933	Low	M	87	MCI	ADNC, low	9.7	X4	L	MFG
P2933	Low	M	87	MCI	ADNC, low	9.7	Z2	L	ITG
P2474	Intermediate	F	87	MCI	Braak IV	9.5	Z5	L	MFG
P2474	Intermediate	F	87	MCI	Braak IV	9.5	X7	L	ITG
P2508	High	M	82	AD	AD	6.7	Z6	R	MFG
P2508	High	M	82	AD	AD	6.7	Z7	R	ITG
P2592	High	M	86	AD	AD	8.6	X4	L	MFG
P2592	High	M	86	AD	AD	8.6	Z3	L	ITG
P2701	High	F	76	AD	AD	18.0	X4	L	MFG
P2701	High	F	76	AD	AD	18.0	Z1	L	ITG
